# Psychometric network analysis reveals how sensory processing relates to self-reflection traits in adolescence

**DOI:** 10.1371/journal.pone.0335259

**Published:** 2025-12-01

**Authors:** Lisa Raoul, Marie-Hélène Grosbras

**Affiliations:** Laboratoire^‌‌^ de Neurosciences Cognitives, Aix Marseille Université, CNRS, Marseille, France; Federal University of Paraiba, BRAZIL

## Abstract

While recent research links sensory processing to mental traits, this has scarcely been explored in adolescence, a period characterized by changes in self-reflection and onset of mental disorders. This study aims to fill this gap using psychometric network analyses to examine how sensory processing characteristics (somatosensation and interoception) relate to self-reflection traits (tendency to examine one’s body, thoughts, and one’s relation to others) in youths aged 10–25 (N = 816) and whether these associations change with sex and age. Results revealed an interconnected network of sensory and self-reflection variables, organized around three main communities. A first showed that elevated social anxiety is associated with heightened sensitivity to somatosensation; a second that positive reflection towards body appearance positively relate to confidence in interoceptive sensations; a third that reflection towards one’s thoughts (private self-consciousness) linked interoceptive awareness with reflection related to others’ thoughts. Some of these associations between sensory and self-reflection traits are stronger in girls and late adolescence, but results regarding age effects were inconsistent. These findings highlight the need to integrate sensorial aspects into our understanding of adolescents’ psychology.

## 1. Introduction

Adolescence is a critical period for mental health, as many mental disorders emerge during this stage, with some studies indicating that one in five individuals is affected to a degree that requires treatment [1]. A common feature of many of these disorders involves maladaptive self-reflection [2–6]. Self-reflection can be defined as the cognitive tendencies to engage in a conscious examination about one’s thoughts and feelings, one’s own body appearance and one’s social relations and actions [6]. The typical distribution and interrelation of self-reflection traits during adolescence, how they change over time, and interact with other factors, is scarcely studied. This would however provide a benchmark to assess pathological vulnerability, allow orienting education and mental health prevention programs, and allow progressing towards a more integrated picture of adolescents’ self-perception.

Specifically, while a growing body of literature shows that, in adults, sensory processing plays a pivotal role in mental states and traits [7], to our knowledge, how self-reflection traits are linked to sensory processing characteristics has scarcely been studied in adolescence. Sensory processing refers to the detection and interpretation of sensory information across multiple sensory channels [8]. Interindividual differences pertain to differences in sensitivity threshold and/or behavioral response to sensory input [8], as well as awareness and affective response to internal sensations [9,10]. Here, we examined sensory processing characteristics regarding two modalities important for building a cohesive sense of self and relevant in the context of mental health and self-reflection research: firstly, somatosensation, which encompasses information related to touch and movement, mediating the interaction between one’s body and the external world; secondly, interoception, which concerns inputs originating from within the body [11].

In adults, self-reported somatosensory sensitivity has been linked to personality traits such as shyness and fearfulness [12]. Additionally, somatosensory processing difficulties are a transdiagnostic phenotype of mental disorders [13]. They have been observed, for example, in autism spectrum disorders [14,15], attention deficit disorder [16], schizophrenia [17] and anorexia nervosa [18–20], including in children and adolescents [21–25]. Interoceptive sensitivity refers to the self-reported tendency to recognize and pay attention to internal body states [26]. In healthy adults, attention to, and reliance on, interoceptive sensations have been found to be associated with emotional regulation, identification of emotions in self and others [27–29] and perspective taking abilities [27]. An interoceptive deficit, that is difficulties in perceiving internal body signals, has been linked to anorexia nervosa [30–32]. In contrast, atypically high noticing of interoceptive sensations has been associated with panic disorder and anxiety [33]. In adolescents (12–15 years-old) interoceptive deficits have been shown to predict eating disorder symptoms and suicidal ideation observed 6 months later [34], pointing to the need to explore further this dimension at this age.

During adolescence, self-reflection traits undergo major changes. For example, from the beginning to middle of adolescence, body esteem decreases drastically [35–37]. Mid-adolescents (13–17 years old) are also more influenced by peers than younger or older individuals [38]. This is happening concomitantly with important somatosensory changes occurring due to puberty and growth spurt [39,40]. The links between sensory processing and self-reflection traits are thus likely to be specific and to change during this period. For example, Murphy *et al.* [41] proposed that the increased prevalence of alexithymia during mid-adolescence could be linked to an altered interoceptive sensitivity specific to this period of life. It has also been proposed that, with increasing age and experience, individuals may adapt better to sensory changes, and mental traits would become less impacted by sensory processing.

Some studies showed that women and men differ in the way they experience bodily sensations, as well as in some self-reflection traits [42,43]. Studies that report a link between sensory processing and psychological traits or mental disorders often disregard these disparities. Neglecting to consider this factor provides a distorted picture [44] and may result in educational and healthcare programs failing to offer gender-responsive services. We primarily investigate the effect of the variable sex, but also asked about gender, that is the person self-representation as male or female, which is also shaped by environmental and social factors. By examining differences between cisgender and non-cisgender participants (e.g., those who report being female in terms of sex but do not identify as girls in terms of gender), we aim to highlight potential effects specifically related to social factors.

Our first aim was to identify how sensory processing characteristics relate to self-reflection traits in a population of adolescents. To do so, we used psychometric network analysis, an approach that offers fine granularity and a holistic understanding of complex, multi-layered relationships among numerous variables. This approach has gained traction over the past two decades in mental health research conceptualizing mental disorders as networks of co-occurring symptoms that influence and reinforce one another [45]. It has also been used in research on personality and traits, contending that personality and traits may be better represented as emergent properties of complex networks of interconnected observed variables, rather than as underlying latent variables (e.g., dimensional personality factors). Psychometric networks are made of nodes representing psychological variables and edges representing statistical associations (e.g., partial correlations) between pairs of nodes [46]. Unlike factor analysis, which aims to reduce data by identifying latent constructs, psychometric network analysis typically focuses on identifying patterns of association among variables without reducing their number [46]. Psychometric network analysis is particularly well-suited to examining the multidimensional factors associated with mental health or personality [47]. The intuitive graphical representation can aid understanding complex relations. One possible extension is *community detection*, where researchers identify sets of nodes that have more connections within the set than between the sets [48,49]. Community detection thus helps making conceptual links between subsets of the observed variables. *Centrality,* which depicts how strongly a node is connected within the entire network, regardless of which community it belongs to, allows identifying the most central or influential nodes, while *bridge strength* can reveal nodes acting as bridges across communities, like, in our case, connecting self-reflection and sensory processing features [50]. In addition, network of interrelations among multivariate constructs can be compared across different groups [51]. We hypothesized a network integrating both sensory and self-reflection nodes, with communities encroaching on both domains and nodes linking the two domains, rather than a strict segregation.

Secondly, we aimed to investigate whether the network differs between early and late adolescence, as well as according to sex. We hypothesized that the period from beginning to mid-adolescence (ages 10–17) would show a stronger association between sensory processing characteristics and self-reflection traits compared to late adolescence and emerging adulthood (ages 18–25). Given the limited number of studies addressing sex differences, we did not formulate specific predictions in this respect.

Regarding sensory characteristics, we focused on commonly used self-reports on the perception and reaction to somatosensory and interoceptive stimuli. Regarding self-reflection traits, we focused on traits particularly relevant during adolescence and that, when pronounced, may predispose individuals to mental disorders. We employed scales and subscales previously validated in adult and, when possible, in youth populations, and shown to reflect specific constructs, with relevant to different aspects of body- or self -reflection. We examined reflections oriented towards body esteem for appearance, weight, and the attribution of others’ opinions, as well as felt pressure from peers, family, and media. We assessed reflection towards the mental self as private self-consciousness, characterizing reflections oriented towards our own thoughts and mental functioning. Finally, we quantified different aspects of reflection oriented towards the social aspect of self and others, including public self-consciousness (adopting a third-person perspective on oneself), empathic concern, perspective-taking, and resistance to peer influence. This multidimensional approach allows us to question traits that are related yet distinct.

## 2. Materials and methods

### 2.1. Participants

We collected data from 816 individuals aged 10–25 years who completed an online survey between June 2021 and May 2023 (See S1 File for information about recruitment). Inclusion criteria were to be between 10–25 years and to be fluent in French. Information about the goal of the study was provided in an online form accessible by the participants, and their legal guardians for minor participants. They had to read this information and consent to participation, by ticking a box, before entering the survey. No identifying data was collected. The sampling design was based on convenience and voluntary participation, which may introduce self-selection biases. We attempted to mitigate this by recruiting across various regions and settings (e.g., schools, sports clubs, online platforms), aiming for a broad and diverse representation of adolescents in France. Of those 816 datasets, we excluded 75 from participants who answered “I do not wish to respond” on 16 or more items (10%). Data from 7 subjects were also excluded because free comments or erratic response patterns indicated non-sincere responses, or because they were outliers in terms of puberty development. The final sample included 734 participants (505 girls). We did not conduct a formal a priori power analysis; however, the sample size is consistent with that of similar previously published studies (e.g., [30]). Detailed demographic characteristics of the final sample are presented in Tables 1 and 2. The study was conducted in conformity with the declaration of Helsinki and approved by the local ethics committee (IRB reference number: 2021-05-06-09); data was handled in conformity with the French regulation and European General Data Protection Regulation.

**Table 1:** Descriptive statistics and internal consistency measure of the continuous measures.

Measures	Minimum	Maximum	Mean	SD	skew	kurtosis	C. alpha
Age	121.00	303.00	206.63	39.82	0.00	2.21	
BMI	12.33	41.38	21.35	3.79	1.05	5.42	
Puberty	1.00	4.00	3.25	0.70	−0.97	3.24	
Puberty compared to age	−1.50	1.40	0.00	0.42	−0.51	3.64	
Private Self-Consciousness	1.38	5.00	3.66	0.71	−0.51	2.95	0.78
Public Self-Consciousness	1.00	5.00	3.53	0.88	−0.43	2.62	0.84
Social Anxiety	1.00	5.00	3.15	0.95	−0.16	2.35	0.79
Perspective Taking	1.00	5.00	3.38	0.72	−0.27	2.78	0.65
Empathic Concern	1.33	5.00	3.86	0.75	−0.68	3.29	0.78
Behavioral engagement	1.00	5.00	3.91	1.19	−1.11	3.25	0.90
Cognitive engagement	1.00	5.00	2.62	1.11	0.21	2.07	0.74
Affective engagement	1.00	5.00	2.40	1.01	0.39	2.41	0.76
Body Esteem Appearance	1.00	5.00	3.22	0.90	−0.31	2.43	0.89
Body Esteem Attribution	1.00	5.00	3.00	0.72	−0.05	3.22	0.55
Body Esteem Weight	1.00	5.00	3.33	1.11	−0.43	2.12	0.92
Family Pressure	1.00	5.00	2.46	1.07	0.45	2.34	0.75
Peer Pressure	1.00	4.75	2.19	0.86	0.43	2.46	0.62
Media Pressure	1.00	5.00	3.16	1.05	0.01	2.13	0.70
Noticing body sensations	1.00	5.00	3.44	0.81	−0.35	3.00	0.67
Not-Distracting	1.00	5.00	2.71	0.95	0.16	2.51	0.65
Not-Worrying	1.00	5.00	2.99	0.91	−0.08	2.54	0.55
Emotional Awareness	1.00	5.00	3.43	0.94	−0.43	2.65	0.79
Body Listening	1.00	5.00	2.60	1.02	0.37	2.51	0.77
Body Trusting	1.00	5.00	3.41	1.02	−0.32	2.51	0.79
Social Touch	1.00	4.71	2.86	0.80	−0.14	2.43	0.73
Sensitivity Threshold	1.57	4.64	3.27	0.52	−0.22	2.91	0.68
Sensitivity Behavior	1.56	4.67	3.41	0.54	−0.11	2.74	0.44
Resistance to Peer Influence	1.00	4.00	3.09	0.61	−0.94	4.30	0.68

**Table 2:** Descriptive statistics of the ordinal measures.

Measures	Level.1	Level.2	Level.3	Level.4	Level.5
Sex. N (1 = Girl)	N = 505	N = 229			
Sex. Freq	0.69%	0.31%			
Cisgender. N (1 = Cis)	N = 27	N = 707			
Cisgender.Freq	0.04%	0.96%			
Physical. Activity N (1 = low)	N = 312	N = 126	N = 172	N = 124	
Physical Activity.Freq	0.43%	0.17%	0.23%	0.17%	
Chronic Pain N (1 = 0)	N = 502	N = 89	N = 78	N = 36	N = 29
Chronic Pain Freq	0.68%	0.12%	0.11%	0.05%	0.04%
Economic Status N (1 = low)	N = 56	N = 182	N = 376	N = 120	
Economic Status Freq	0.08%	0.25%	0.51%	0.16%	

### 2.2. Measurements

The survey comprised 163 items, from which we obtained 32 measures. Measures related to sensory processing characteristics included: chronic pain, interoceptive awareness (body noticing, not-distracting, not-worrying, listening and trusting and emotional awareness of the body), sensorimotor sensitivity (sensitivity threshold and behavior to sensory stimulation) and social touch. Measures related to self-reflection traits included body-esteem for appearance, for weight and attribution; family, peers and social-media appearance-related pressure; private and public self-consciousness and social anxiety; perspective taking and empathic concern; and resistance to peer influence. We also collected general information about personal and lifestyle features. All scales were validated psychometric scales, except three subscales constructed based on questions from validated scales (social touch, sensitivity behavior, and sensitivity threshold). Due to legislation, we could not collect data on race and ethnicity. Additional information about the instruments is provided in S1 File.

#### 2.2.1. Personal and life-style variables.

Participants were asked to provide information about their *Age*, height, and weight. *Body Mass Index* (BMI) was calculated from the weight/height ratio. They were asked to indicate, in separate questions, their *Sex* and Gender, by selecting Male, Female or Other, with also the option “Do not wish to reply”. Using this information, we derived a binary variable indicating whether the participant was *Cisgender*, that is whether their gender identity matches the sex they’re identified with. As a proxy for *Subjective Economic Status*, participants were asked to rate the economic situation of their family/environment during their upbringing on a 5-points scale (“very difficult”, “difficult”, “neither difficult nor comfortable”, “comfortable”, “very comfortable”). Only three participants reported the first level, which was thus combined with the second.

From the Petersen Pubertal Scale [52], we derived an index of *late or early-puberty*, by comparing each individual score to the function puberty *vs* age estimated from our sample (see S1 File). We also asked about *Physical activity*, based on four questions [53], and engagement in *Social Media (affective, cognitive and behavioral)* [54] (see S1 File).

#### 2.2.2. Sensory processing characteristics.

**2.2.2.1. *Chronic pain:*** Participants were asked to report the number of *Chronic pain* sites with episodes of pain lasting three months or more, as an index of central sensitivity to nociception.

**2.2.2.2. *Sensitivity threshold and behavior to somatosensory stimulation:*** We used 23 items from the Brown and Dunn’s Adult and Adolescents Sensory Profile questionnaire [55] to characterize *sensitivity threshold* (high or low threshold) and *behavioral response* to sensory stimulation (sensation seeking or avoiding). Items of both measures relate to sensory processing of touch and movement (the original version covers also taste, smell, vision, and audition). The Likert scales ranged from “nearly never” (1) to “nearly always” (5), with higher scores indicating a high threshold (i.e., low sensitivity to somatosensory sensations) and sensation seeking.

**2.2.2.3. *Social Touch:*** We used a 7-items questionnaire derived from the *Social Touch* Questionnaire [56] and from the Dunn Sensory Profile [55] to evaluate the extent to which individuals appreciate touching and being touched in a social context. The Likert scales ranged from “nearly never” (1) to “nearly always” (5), with low scores meaning strong avoidance of social touch.

**2.2.2.4. *Body noticing, not-distracting, not-worrying, listening and trusting and emotional awareness of the body*:** We used six subscales of the French version of Multidimensional Assessment of Interoceptive Awareness (MAIA) [9] validated in youth [10]. *Noticing* concerns the subjective awareness of body sensations. *Not-Distracting* investigates how often a person tends not to ignore sensations of pain or discomfort. *Not-Worrying* assesses the extent to which a person doesn’t worry about, nor catastrophizes, discomfortable sensations. *Emotional Awareness* deals with the awareness of the relation between emotional and bodily states. *Body Listening* assesses how often a person actively attends to their bodily sensations for insight. *Body Trusting* investigates the extent to which a person experiences their body as a safe and trustworthy source of information. The Likert scales ranged from “never” (1) to “always” (5), with higher scores indicating greater awareness, listening and trust in one’s body, and reduced worrying.

#### 2.2.3. Self-reflection traits.

**2.2.3.1. *Body-esteem for appearance, for weight and attribution:*** We used the French version [57] of the Body-Esteem Scale validated for adolescents [58]. The questionnaire consists of three subscales. The *Appearance* subscale assesses general feelings about one’s looks. The *Weight satisfaction* investigates perception of one’s own weight and the extent to which one would like to change it. The *Attribution* subscale assesses beliefs about how others perceive one’s appearance. The Likert scales ranged from “never” (1) to “always” (5), with higher scores indicating more positive feelings about appearance, greater weight satisfaction, and positive beliefs about how others perceive one’s body.

**2.2.3.2. *Family, peers and social-media appearance-related pressure:*** We used the French version [59] of the three subscales from the Sociocultural Attitudes Towards Appearance Questionnaire 4 (SATAQ-4) [60] focusing on appearance-related pressure. These scales assess pressures from *Family*, *Peers*, and *Social Media*. The Likert scales ranged from “definitely disagree” (1) to “definitely agree” (5), with higher scores indicating greater felt pressure. Following reports of misunderstanding in pilot tests, we modified the wording of four items.

**2.2.3.3. *Private self consciousness, public self consciousness and social anxiety:*** We used the 22-items French version [61] of the revised version [62] of the Self Consciousness Scale (SCS) [63]. The SCS consists of three subscales. The *Private Self-Consciousness* subscale assesses the extent to which individuals focus on internal thoughts and analyze their own mental functioning. The *Public Self-Consciousness* subscale quantifies the extent to which an individual tends to focus on oneself as a social agent that can be observed and judged by others. The *Social Anxiety* subscale measures distress caused by interacting with others. The Likert scales labels ranged from “extremely uncharacteristic of me” (1) to “extremely characteristic” (5), with higher values indicating a higher tendency to focus on oneself/ be anxious.

**2.2.3.4. *Perspective taking and empathic concern:*** We used two subscales of the French version [64] of the Interpersonal Reactivity Index (IRI) [65], commonly used in adolescents populations [66]. *Perspective Taking* assesses the tendency or ability to adopt the perspective of other people and is considered a measure of cognitive empathy. *Empathic Concern* assesses the tendency to experience feelings of warmth, compassion, and concern for others undergoing negative experiences and is considered a measure of affective empathy. The Likert scale displays ranged from “this doesn’t describe me at all” (1) to “this describes me very well” (5), with higher values indicating higher empathy.

**2.2.3.5. *Resistance to peer influence:*** The Resistance to Peer Influence (RPI) Scale [38] consists of 10 pairs of statements (i.e., ‘’some people… but other people.”). Participants are asked which of the two statements describes them better. Responses are then coded on a 4-point scale, ranging from “really true” for one descriptor to “really true” for the other descriptor, and averaged. Higher scores indicate greater resistance to peer influence.

### 2.3. Data analyses

Missing data handling are presented in S1 File.

#### 2.3.1. Overlap analysis: Assessing discriminant features of the various measures.

We checked for topological overlap between the different measures using the Goldbricker function from the R package *networktools* [67] (version 1.6.0) with a threshold of 0.35, and the default “hittner2003” method. This recommended procedure [47,68] checks for pairs of variables that have similar connections to other variables in order to identify variables that most likely measure the same underlying construct [67]. Such overlap would influence the network architecture [69]. The function’s output provides an indication of which nodes to remove prior to network estimation. Because of excessive overlap, we removed two subscales for the network analysis: the *Body Esteem for Weight* (67% overlapping correlations with *Body Esteem for Appearance*) and *Noticing of body sensations* (77% overlapping correlations with *Emotional awareness of the body*). The final network was therefore made of 30 measures. We applied the same procedure with a threshold of 0.25 before estimating the networks of each group that we compared (see below). For the group of the male participants *Emotional Awareness* was removed (88% overlapping correlations with *Body Listening*) and therefore also removed for the group of girl participants during network comparison between sexes. *Behavioral Engagement in Social Media* was excluded in young girls due to high overlap with *Affective Engagement* (87%) and was also removed in older girls to ensure comparability across groups.

#### 2.3.2. Network estimation.

To study the complex, fine-grained interconnections among the variables, we conducted a network analysis in R (version 4.4.1) using the *bootnet* (version 1.6) and *qgraph* (version 1.9.8) packages [70]. Based on the covariance matrix of the 30 variables, we used the estimateNetwork() function to estimate partial correlation networks, which model the unique association between each pair of variables while controlling for all others. We then visualized the results using qgraph().

First, we estimated a Gaussian Graphical Model (GGM) using the least absolute shrinkage and selection operator (LASSO) with Extended Bayesian Information Criterion (EBIC) model selection (with the default hyperparameter γ = 0.5) [71,72]. This approach, known as EBICglasso, is widely used in psychological network research for datasets with a similar number of nodes and sample size. The resulting network was relatively dense, reflecting high sensitivity, but potentially lower specificity.

To address this potential lack of specificity, we repeated the EBICglasso estimation with an additional, global, thresholding procedure, removing edges with value in the precision matrix below log(p*(p-1)/2)/ sqrt(n), with n being the number of participants (734) and p the number of variables (30) [70].

We also estimated the network using the ggmModSelect algorithm implemented in *bootnet*. This is a fast non-regularized algorithm based primarily on the glasso algorithm in combination with BIC model selection [73]. For sample sizes and network dimensions comparable to ours, this method has been shown to yield lower sensitivity but higher specificity for both edges and bridge edges [73]. This makes it a valuable complementary approach for identifying the most stable and interpretable network connections.

Finally, based on the findings of Williams and Rast [74], who showed that unregularized network estimation performs well for this sample size and number of nodes, we also estimated unregularized partial correlation networks, applying false discovery rate (FDR) correction to control false positives.

We selected the EBICglasso without thresholding as our main network estimation procedure for subsequent analyses. This method remains the most widely used in psychological network literature and is well-suited for identifying overall network structure and strong associations at our sample size [73]. To assess whether the identification of the most prominent edges depended on the estimation procedure, we repeated all key analyses using the alternative estimation methods. Results were largely consistent across models, with only minor variations in network structure. These complementary analyses are reported in the S1 File and confirm that our main conclusions do not depend on the choice of estimation procedure.

Additionally, to address potential concerns regarding collider bias or spurious associations, we compared the zero-order and partial correlation matrices. This analysis confirmed that the identified edges were not a byproduct of considering too many variables. Full details are provided in the S1 File.

#### 2.3.3. Centrality and bridge estimation.

For each network obtained using the *bootnet/qgraph* packages, we looked at strength centrality, using the centrality function in the R package *qgraph*, and bridge-strength centrality using the bridge function in the R package *networktools*. Strength centrality is the sum of the absolute weights of all edges connected to a node and is a measure of its overall connectedness. Other centrality indices, such as closeness and betweenness, could be used, but have been shown to be unstable [75]. Likewise, expected influence overlooks negative edges, which is not compatible with our study, as score directions vary across scales (higher scores meaning more sensory processing or self-reflection in some, and the opposite in others) and negative edges may carry different meanings. Bridge strength quantifies the extent to which a node within a specific community (i.e., a group of nodes representing the same psychological domain) connects to nodes in other communities. In the present study, bridge strength was calculated to identify sensory nodes with the strongest connections to self-reflection nodes, and conversely, self-reflection nodes most strongly connected to sensory nodes. Bridge strength is computed as the sum of the absolute weights of a node’s edges that connect it to nodes in other communities. We emphasized bridge strength because centrality strength have been shown to be sensitive to the specific composition of nodes within a network, which may limit their interpretability in psychometric network analyses [75]. In contrast, bridge indices are invariant to the type of network estimated, rendering them especially useful in psychometric research. We qualified as bridge-node, nodes with a bridge strength larger than one standard deviation above the mean bridge strength.

#### 2.3.4. Communities estimation.

For each network estimated with the *bootnet/qgraph* packages, we applied Exploratory Graph Analysis (EGA) to identify communities—that is, groups of nodes showing similar patterns of association with the rest of the network [48]. We used the community.detection() function from the *EGAnet* package (version 2.3.0) with the argument algorithm = “optimal”. This analysis was conducted to test whether the detected communities aligned with our predefined theoretical categories, namely self-reflection and sensory processing.

#### 2.3.5. Network comparison between groups.

We investigated the differences due to sex or age in our dataset. Networks were estimated for all subgroups using the same procedure as for the whole sample. The obtained networks were then compared using permutation tests implemented in the NCT() function of R package *NetworkComparisonTest* (NCT) [51] (version 2.2.2; 2000 permutations). For each of the two comparisons (between sexes and between age-groups), we compared the networks in terms of global structure, global strength, individual edges that link one node of sensory processing with one related to self-reflection, strength centrality, and bridge-strength measures. When the global structure and global strength are not significantly different between two networks, differences in individual edge weights in these groups are more likely to be of a content-related nature and not due to noise/measurement error [76]. Due to the conservative nature of the NCT [51], we report results both with and without a Bonferroni-Holm correction for multiple comparisons. We applied this family-wise error correction considering only the edges that link one node of sensory processing with one related to self-reflection. In addition, similarly to previous work [76], we calculated difference networks by subtracting the weights of one network from the corresponding weights of the other network.

#### 2.3.6. Robustness analysis.

Standard robustness analyses were conducted using case-dropping bootstraps to evaluate the stability of edge weight estimates, differences between edges, centrality, bridge strength, and community structure. These analyses were applied to all networks estimated using the EBICglasso regularization method without thresholding—both the main network on the full sample and the four subgroup networks used for group comparisons. Detailed results are provided in the S1 File.

## 3. Results

### 3.1. Descriptive statistics

Tables 1 and 2 show the descriptive statistics and internal consistency (Cronbach’s coefficient alpha) for each of the variables used as node in the network for the whole sample (see S1 File for this information for the different groups).

In S1 File we report the zero-order pairwise correlation matrix between these variables and contrast it with the partial correlation matrix, highlighting differences in the strength and structure of associations.

### 3.2. Whole sample network estimation

**Fig 1:**
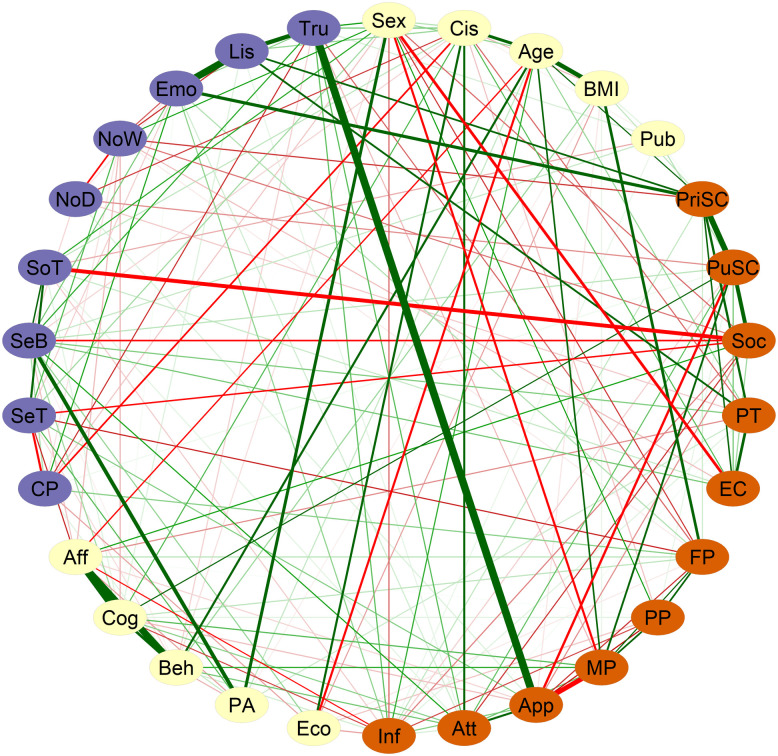
Network of relations between self-reflection traits, sensory processing characteristics, and other covariates in adolescent boys and girls (n = 734) as estimated with the EBICglasso regularization method without global threshold. Each node represents a measure, and each link represents the partial correlation between two nodes. Orange nodes represent self-reflection traits, purple nodes represent sensory processing characteristics, and white nodes represent covariates. The thickness of the edges indicates the strength of the correlation, and the color indicates its sign (positive = green, negative = red). See Fig 2 for node descriptions.

**Fig 2:**
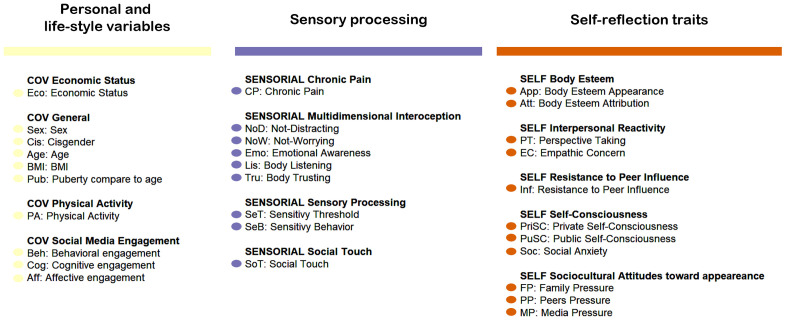
List of variables used as nodes in the networks.

Fig 1 shows the network obtained using the EBICglasso regularization method (with labels displayed on Fig 2). This analysis, characterized by high specificity, indicates that the edges across domains most likely to exist reflected (1) a strong positive correlation between body trusting trait and the body appearance domain of body esteem (r = 0.39), (2) a negative correlation between the level of social anxiety and sensitivity behavior (r = −0.13) and appetence for social touch (r = −0.23), and (3) a positive relation between emotional awareness and private self-consciousness (r = 0.20), between body listening and private self-consciousness (r = 0.14), and between body listening and perspective taking skills (r = 0.1).

Personal and lifestyle factors (age, pubertal status or engagement with social media) were mainly linked to measures of self-reflection and not sensory processing. Robustness analyses are present in S1 File.

**Fig 3:**
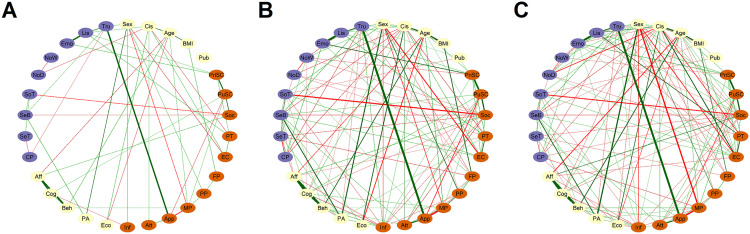
Networks of relationships between self-reflection traits, sensory processing characteristics, and other covariates in adolescent boys and girls (n = 734), estimated using (a) the EBICglasso regularization method with additional global thresholding (promoting higher specificity), (b) the ggmModSelect method, or, (c) partial correlations corrected for multiple comparisons using FDR. For all three networks, the minimum visualization threshold was set to 0 and the maximum to 0.7.

We replicated the main analyses using three alternative network estimation methods: a thresholded version of EBICglasso, the ggmModSelect algorithm [73], and unregularized partial correlations with False Discovery Rate (FDR) correction [74]. Although the exact network structures varied slightly, the strongest edges, overall community patterns, and bridge nodes remained largely consistent across models (see Fig 3). More detailed results are presented as S1 File.

### 3.3. Centrality indexes

**Fig 4:**
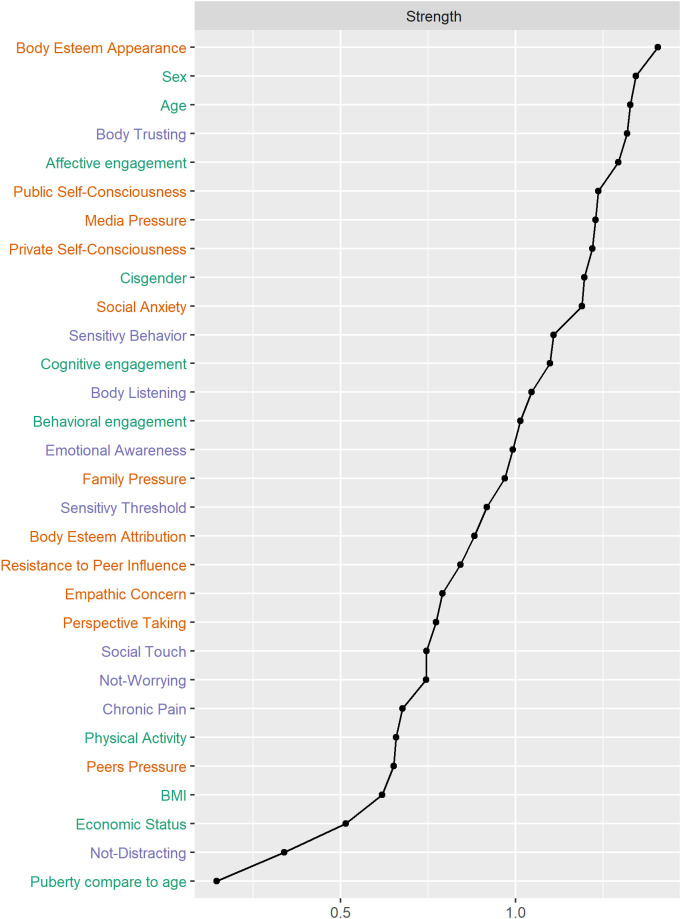
Strength centrality for all nodes in the network estimated with EBICglasso regularization (sensory-processing characteristics (purple), self-reflection traits (orange), and covariates (green).

**Fig 5:**
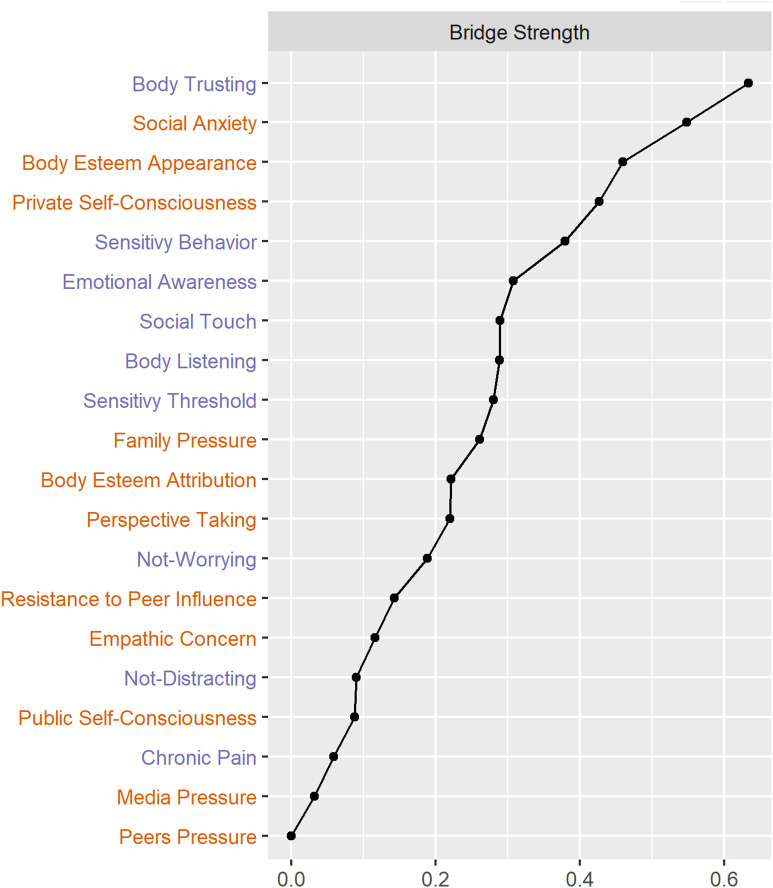
Bridge strength index for sensory-processing characteristics (purple) and self-reflection traits (orange). Bridge centrality examines the extent to which each sensory-processing characteristic node is connected to all self-reflection nodes, and vice versa. Four nodes had bridge centrality values greater than one standard deviation above the mean and are therefore identified as bridge nodes: Body Trusting, Social Anxiety, Body Esteem – Appearance, and Private Self-Consciousness.

Centrality strength values are presented in Fig 4 and bridge strength values are reported in Fig 5. Four nodes had bridge strength values greater than one standard deviation above the mean (i.e., greater than 0.424): body trusting (0.634), social anxiety (0.548), body esteem appearance (0.459), and private self-consciousness (0.427). Robustness analyses are present in S1 File.

Bridge centrality examines the extent to which each sensory-processing characteristic node is connected to all self-reflection nodes, and vice versa. Four nodes had bridge centrality values greater than one standard deviation above the mean and are therefore identified as bridge nodes: body trusting, social anxiety, body esteem – appearance, and private self-consciousness.

### 3.4. Community analyses

**Fig 6:**
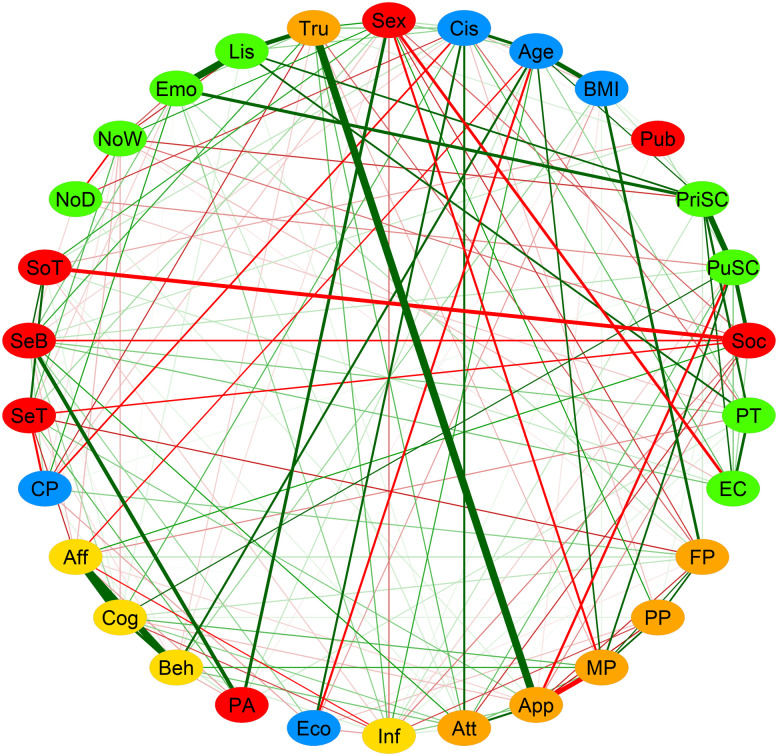
Communities resulting from the community detection of Exploratory Graph Analysis applied on the network presented in Fig 1. The network is divided into five communities, with three communities (orange, red, green) integrating sensory processing nodes and self-reflection nodes, and two communities (blue and yellow) consisting mostly of covariates. Each node represents a measure, and each link represents the partial correlation between two nodes. Here, the color of the nodes represents the community to which each measure belongs. The thickness of the edges indicates the strength of the correlation, and the color of the edges indicates the sign of the correlation (positive = green, negative = red). See Fig 2 for node descriptions.

The results of the community analysis using Exploratory Graph Analysis are presented in Fig 6. Five distinct communities emerged. The first community (red) included social anxiety, sensitivity threshold, sensitivity behavior, social touch, and physical activity as well as sex. The second community (orange) comprised body trusting and variables related to body appearance, including, body esteem appearance, body esteem attribution and perceived pressure from media, peers, and family regarding appearance. The third community (green) included private and public self-consciousness, the two subscales of the interpersonal reactivity index (perspective taking and empathic concern), along with the remaining nodes related to interoceptive awareness (not-distracting, not-worrying, emotional awareness, and body listening). The fourth community (blue) included age, BMI, economic status, and gender. The fifth community (yellow) comprised resistance to peer influence along with the behavioral, cognitive, and affective dimensions of social media engagement. Robustness analyses are provided in S1 File.

### 3.5. Networks comparisons

We investigated the differences due to sex and age. In the collected dataset there were only 130 older boys and 100 younger boys. This was not enough to achieve sufficient network stabilities and compare these two groups. Therefore, we investigated age differences only among female participants.

#### 3.5.1. Comparison between sexes.

**Fig 7:**
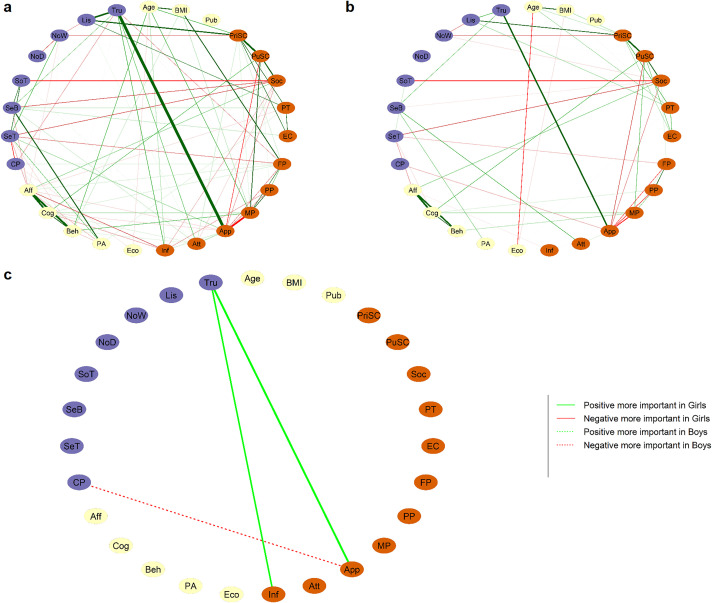
Comparison between sexes. Networks for adolescent girls (a) and boys **(b)**. Edge width is scaled like for the whole sample with a maximum of 0.7 and a minimum of 0. In **(c)**, the difference between networks a and b is computed and only edges that are significantly different are plotted. Edge thickness is scaled to the absolute value of r difference (plotted with argument cut = 0.001). Edge colors and line types represent group differences as follows: Green solid lines = Positive edges stronger in girls. Red dotted lines = Negative edges stronger in boys. See Fig 2 for node descriptions.

Networks for girls and boys are presented in Fig 7a and 7b. Global strength comparison tests showed that the difference between the girls network’s global strength (3.51) and the boys network’s global strength (2.06) was not significant (Test statistic S: 1.45, p = 0.31). The network invariance test was also not significant (Test statistic M: 0.18, p = 0.47). Since the overall level of connection was not significantly different between the two groups, differences in individual edge-weights were more likely to be of a content-related nature.

When not correcting for multiple comparisons, three edges linking a sensory node with a node related to self -reflection traits were found to be significantly different between boys and girls (Fig 7c). The positive connections between body trusting and body appearance esteem (p = 0.027) and between body trusting and Resistance to peer influence (p = 0.019) were stronger in girls. The negative connection between chronic pain and body esteem for appearance was stronger in boys (p = 0.037). Body trusting (p = 0.013) and resistance to peer influence (p = 0.031) displayed bridge-strength higher in girls compared to boys. No significant difference was found regarding strength centrality. When applying Bonferroni-Holm correction for 88 multiple comparisons, there were no statistically significant edge, centrality-strength, or bridge-strength differences.

The Network Comparison Test on networks estimated with alternative methods (see S1 File) showed some variations, but body trusting and body esteem appearance consistently emerged as bridge nodes with stronger connections in girls than in boys.

#### 3.5.2. Comparison between age-groups in girls.

**Fig 8:**
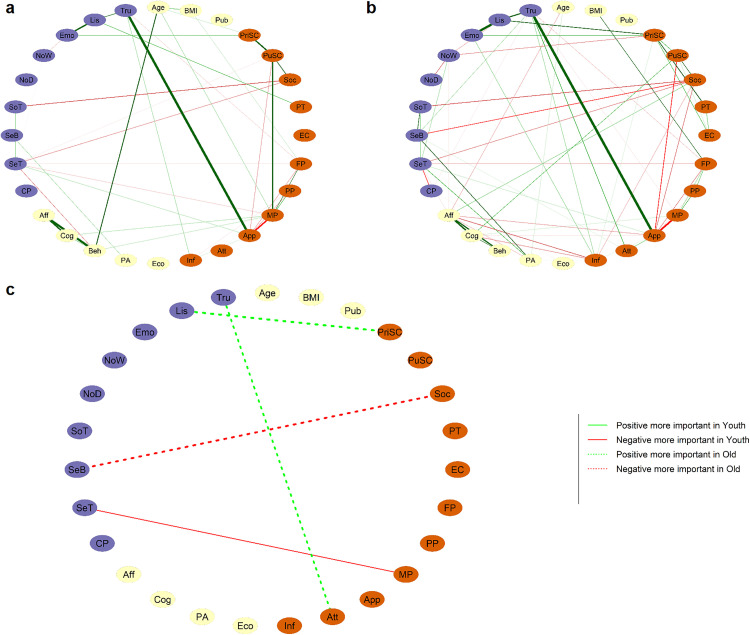
Comparison between age-groups in girls. Networks for younger girls (a) and older girls **(b)**. In **(c)**, the difference between networks a and b is computed and only edges that are significantly different are plotted. Edge thickness is scaled to the absolute value of R difference (plotted with argument cut = 0.001). Edge color and style indicate group differences: Red solid lines = Negative edges stronger in younger girls. Green dotted lines = Positive edges stronger in older girls. Red dotted lines = Negative edges stronger in older girls. See Fig 2 for nodes description.

Networks for younger girls and older girls are presented in Fig 8a and 8b. Global strength comparison tests showed that the difference between young girls’ global strength (1.64) and older girls’ global strength (1.85) was not significant (Test statistic S: 0.20, p = 0.843). The network invariance test was also not significant (Test statistic M: 0.19, p = 0.35).

When not correcting for multiple comparisons, four edges linking a sensory node with a node related to self-reflection were found to be significantly different between the two groups (Fig 8c). The positive connections between body trusting and body esteem attribution (p = 0.043) and between body listening and private self-consciousness (p = 0.036) were stronger in older girls. The negative connection between sensitivity behavior and social anxiety was stronger in older girls (p = 0.039). The negative connection between sensitivity threshold and media pressure (p < 0.001) was stronger in younger girls. No significant differences were found regarding strength centrality and bridge-strength. None of the significant differences remained after applying multiple comparisons correction.

The Network Comparison Test on networks estimated with alternative methods (see S1 File) showed different results regarding global strength, individual edge weight and bridge edges.

## 4. Discussion

Applying network analysis, we explored, in a group of adolescents, the complex relations between self-reported sensory processing characteristics (somatosensory and interoceptive) and traits related to reflections about one’s thoughts, one’s own body appearance and oneself and others in social context, and how these relations may change as a function of sex and age.

Only two scales were removed because they showed significant overlap in their patterns of relations with other measures. This provides further evidence that each of the remaining variables used as nodes in the network targets a specific construct distinct from what is measured by the other variables. Therefore, the different subscales we used allow us to obtain fine-grained information about the relations between distinct self-reflection traits and sensory characteristics.

The network we obtained showed numerous connections between self-reflection traits and sensory processing characteristics. In the whole group, all sensory nodes were related to self-reflection nodes, when the network was estimated with the most sensitive method (EBICglasso without threshold). The most important edges between sensory and self-reflection nodes remained in the highly specific network obtained by applying a global threshold in combination with the EBICglasso method. Community analysis revealed that the network was better described by communities composed of both sensory and self-reflection nodes, rather than by communities composed solely of sensory nodes or solely of self-reflection nodes. The communities and their specific relations would have been less visible through a traditional relational or factorial approach. Bridge centrality analysis revealed body trusting as a bridge node between the sensory processing and the self-reflection categories. It also revealed that social anxiety, body esteem for appearance, and private self-consciousness were bridge nodes that strongly relate to sensory nodes, each belonging to a different community. Below, we discuss specific communities of interactions between components of the two conceptual domains in the whole group, as well as the differences in network structure related to sex and age.

### 4.1. A network integrating sensory processing characteristics and self-reflection traits

We measured interindividual covariation in variables conceptually grouped into three categories: self-reflection traits and sensory processing characteristics, as well as demographic and lifestyle covariates. Yet the network analysis of these variables revealed five communities, three of which integrated sensory nodes and self-reflection nodes, while two were composed of covariates. This demonstrates strong and specific interrelations between the two domains.

#### 4.1.1. Somatosensory processing relates to social anxiety.

The first community (red community in Fig 6) connected social anxiety to three somatosensation nodes, and to physical activity. Social anxiety was also one of the self-reflection traits with high bridge strength. This indicates that social anxiety is related to how adolescents experience and react to somatosensory stimulation. Adolescents with a low threshold (i.e., very sensitive to somatosensory sensations) have a higher tendency to feel anxious in social situations. Previous studies have shown that children and adolescents diagnosed with a general anxiety disorder consistently display a heightened sensitivity to external stimuli [23]. We extend this finding to a non-clinical population and our results support the idea that heightened sensory sensitivity could be a sign of social anxiety vulnerability. Our results also invite future research to test whether somatosensory habituation training could help to prevent anxiety disorders onset.

Additionally, in our data, the tendency to engage in social touch was negatively related to social anxiety and positively related to sensitivity threshold and sensitivity behavior. This extends findings in adults [77]. Despite the present analysis not allowing us to test directionality, we can hypothesize that these relations are bidirectional. Individuals experiencing more anxiety in social interactions are less likely to engage in social touch. Reciprocally, people who engage less in social touch are less likely to appreciate touching and being touched, and may thus be more likely to experience social anxiety. In parallel, social touch has been found to have positive consequences on the psychological and physiological human development [78], and to reduce anxiety [79]. Since, adolescents show a heightened neural response to pleasant touch compared to adults [80], this could be leveraged to break this cycle of reciprocal influences in case of anxiety at this age.

#### 4.1.2. Interoceptive awareness intertwined with reflections on one’s own and others’ thoughts.

Another community (green community in Fig 6) integrated all nodes of the MAIA questionnaire (interoceptive awareness), except body trusting, with private and public self-consciousness and the two nodes of the interpersonal reactivity index (empathic concern and perspective taking). Adolescents who exhibited a higher propensity for introspection regarding their mental processes and feelings were those who were more attentive to bodily sensations and relied on them to assess their emotional states. Recently, Yang *et al.* [43] reported a correlation between MAIA questionnaire scores and self-consciousness scale scores in adults. The authors didn’t report which subscales of either questionnaire weighed more in this relation, however. Altogether, these results extend to adolescence the findings from studies conducted in adults that indicate that selfhood is grounded in interoceptive processing [81].

Interestingly, body listening was also associated with perspective-taking, the latter being related to private self-consciousness. This points towards an interaction between the capacity to process signals originating from within the body and the capacity to switch between self- and others- perspective, as it has already been indicated in adults [27]. Previous developmental models proposed that, in adolescence, the construction of self-knowledge is intertwined with the construction of knowledge of others [82]. Based on our results, we propose that the sensitivity to bodily sensations also intervenes in this construction.

#### 4.1.3. Trusting bodily sensations relates to the tendency to reflect towards one’s body appearance.

A third community (orange community in Fig 6) revealed an association between body trust and nodes related to body appearance. Bridge centrality analysis indicated that body esteem for appearance was strongly linked to sensory nodes, and body trusting was strongly linked to self-reflection nodes. This shows, in a sample of adolescents from the general population, that body trust, that is the perception of information coming from the inner body as reliable, is closely tied to how individuals reflect on their body appearance: young people with low body trust also have negative reflections towards body appearance. This is consistent with what has been shown regarding other facets of body esteem [83] and with previous studies that have shown that low body trust is associated to anorexia nervosa symptoms [30]. In partial correlation networks, links correspond to conditioned associations with other nodes. This means that the link between body trust and body esteem for appearance does not depend on body-mass index, which on its own has been consistently associated with body esteem. At the same time, low body esteem, a psychological trait, is a robust and consistent predictor of anorexia nervosa [84]. These results suggest that preventing body mistrust may reduce the risk of body image disorders, although this must be taken with caution since the links in the network are not directional [85].

### 4.2. Differences between boys and girls

The networks built from boys’ and girls’ data separately showed a highly similar structure, with interrelations between variables from sensory processing and self-reflection. There was no difference in global strength, indicating a similar level of network connectedness for boys and girls; that is, a similar network density. At the edge level, we observed several statistically significant sex-related differences in both edge strength and bridge strength. While results differed slightly by network estimation method, the results consistently indicated a stronger role of body trusting in girls. The link between body trusting and body esteem for appearance, although present in both girls and boys, was stronger in girls, who also showed higher scores for both constructs. The bridge strength of body trusting was also higher in girls compared to boys.

This result should be interpreted with caution, as the sex difference only appeared before correction for multiple comparisons. However, this could suggest that low body esteem in boys relates less to interoceptive sensations, and that body trust is connected to self-reflection in girls, but to a lesser extent in boys. This underscores the necessity of considering sex when examining the role of interoception in body image disturbance and mental health. Since studies on anorexia nervosa predominantly involve girls, their findings may not directly translate to boys. Our results could also have clinical implications: addressing body mistrust might be less effective in mitigating body image issues in boys compared to girls.

To highlight effects specifically related to social factors, we also examined whether individuals whose reported gender did not align with their sex showed different patterns. While a group comparison of networks was not possible, since only 27 participants reported a gender different from their sex, we nonetheless included the cisgender variable in the network analysis. We observed a negative association with sensory characteristics, namely chronic pain and the “not-distracting” component of the MAIA, and a positive association with the body esteem attribution scale. This highlights the need for future research to explore how social factors may influence sensory processing characteristics and their association with self-reflection traits.

### 4.3. Differences between beginning-mid adolescence and end of adolescence-adulthood

Based on a few studies and previous claims, we expected that links between sensory characteristics and self-reflection traits would become weaker during the course of adolescence. Contrary to our expectations, we found only one edge-wise difference in the hypothesized direction and three in the opposite direction. The link between body trust and body esteem attribution was observed in older but not younger adolescent girls, as were the links between body listening and private self-consciousness, and between sensitivity behavior and social anxiety. All three links belonged to a community connecting sensory and self-reflection traits, suggesting that the network observed in the whole sample becomes more clearly expressed towards the end of adolescence. However, these findings should be interpreted with caution, as they did not survive correction for multiple comparisons and results varied across estimation methods. Notably, this pattern diverges from the proposition of Yang *et al.* [43] according to which interoceptive deficits during childhood and adolescence would relapse with age partly thanks to social experience. Instead, our results suggest that interoceptive and somatosensory characteristics may be more related to self-reflection traits in late than in early adolescence. Based on these results, we speculate that the adaptation to sensory changes with experience proposed by Yang *et al.* [43] could occur during the third decade of life, and that the age range of our sample does not allow us to observe this effect. Future research should thus explore this correlation across a wider age spectrum, and possibly with longitudinal design to assess changes more finely as a function of adolescence progression.

### 4.4. Limits and future directions

Firstly, one limitation of the present study concerns the use of voluntary participation and online recruitment, which may introduce self-selection bias. Individuals who chose to take part in the study may differ systematically from those who did not, particularly in terms of psychological openness, digital access, comfort discussing body issues or interest in the topic. Although we attempted to reach a diverse sample by recruiting through schools, sports clubs, and social media across different regions, the final sample remains a convenience sample and may not fully represent all sociodemographic backgrounds.

Secondly, interpreting very weak or missing edges in networks estimated with regularized methods like LASSO or unregularized methods with thresholding (e.g., FDR) is challenging due to the trade-off between sensitivity and specificity. Low-weight edges or absent edges may reflect true weak or absent associations but can also result from penalization and sample variability. Thus, caution is needed before concluding that a relationship is definitively absent based solely on these estimates. For instance, in our network, social anxiety relates only weakly to interoceptive nodes, unlike what might be hypothesized from previous studies [33]. However, this should be interpreted with caution, as given the partial correlation approach and the lasso regularization [71], a putative link could be masked by the fact that most of the inter-individual variability in social anxiety can be explained by taking somatosensory aspects into account (partial correlation effect) and because body trusting highly relates to many others nodes (lasso regularization effect). Other approaches, such as Bayesian statistics, would be needed to confirm the absence of links between social anxiety and interoceptive variables.

As indicated above, the low overlap in patterns of connections of the different nodes indicates that these nodes represent distinct constructs. Yet, a limitation of our study is inherent to the psychometric properties of the variables we entered in the network analysis. Some of the subscales of our questionnaire showed only moderate internal consistency in our sample or/and in the original validation studies. First, we used the first version of the MAIA questionnaire as it contains fewer items, and our questionnaire was already long [9]. However, the consistency of the Not-worrying subscale was low. To improve this, and therefore enhance the reliability of our results, future studies could use the second version of the MAIA, which contains more items and display higher reliability [86]. Consistency was also low for body esteem attribution [58]. Future studies could use more recent scales [87]. Sensitivity Behavior also had a low consistency value. This could be because we used only part of the items from the original scales (as others concern other sensory modalities). Future studies could, therefore, investigate somatosensory processing characteristics using different instruments.

The differences between sexes and age-groups were not statistically significant when correcting for multiple comparisons. Robustness analyses showed acceptable edge stability and edge differences in each group, although these values were lower than for the whole sample network. Therefore, we believe that our approach for group analysis is valid; yet sex and age effects should be tested with larger sample sizes and with a full factorial design allowing for testing interactions between age and sex.

Additionally, for all the dimensions we evaluated, socio-economic status is certainly an important modulator [88]. Here, we could only assess economic status via subjective self-report. This less accurate and more variable measure may have prevented us from uncovering the links that socio-economic status could have with one’s body experience.

## 5. Conclusion

We used network analysis and self-report questionnaires to investigate how sensory processing characteristics relate to reflections about one’s body, one’s thoughts and one’s in relation with others in the general adolescent population, including boys and girls. We uncovered three communities linking sensory processing and self-reflection traits. They showed that somatosensory processing is associated with social anxiety, while interoceptive awareness is integrated with tendencies to reflect on one’s own and others’ thoughts. Finally, trust in interoceptive sensations is associated with attention to body appearance. Our results also suggest that these relations may become stronger at the end of adolescence, which contradict hypotheses of the current literature proposing stronger links at the beginning or middle adolescence. Taken together our findings lend support to the idea that how adolescents feel their body is closely related to the way they experience their body appearance, the self and others. Therefore, when conceptualizing self-development in adolescence, models should also include sensory processing characteristics. More broadly, promotion of mental health in adolescence should consider the intricate relationships between somatosensory and interoceptive processing and higher representations of the self. Our findings open the path to future research testing for causality and longitudinal studies. For instance, one could examine whether teaching adolescents to pay attention to bodily sensations could contribute to the development of social skills or whether stressful social life events predict bodily sensations in later life.

## Supporting information

S1 File Supplementary information. This document contains additional information regarding the psychometric scales used, data preprocessing, detailed results using alternative estimation methods, robustness analyses — namely edge weight estimates, differences between edges, and case-dropping bootstrap —, as well as additional descriptive statistics for subgroups.
(DOCX)

## References

[1] Chabrol H. Les défis de la psychopathologie de l’adolescent aujourd’hui. Traité de psychopathologie clinique et thérapeutique de l’adolescent. Paris: Dunod; 2011. p. 5–35.

[2] Barnes M, Abhyankar P, Dimova E, Best C. Associations between body dissatisfaction and self-reported anxiety and depression in otherwise healthy men: A systematic review and meta-analysis. PLoS One. 2020;15(2):e0229268. doi: 10.1371/journal.pone.0229268 32097427 PMC7041842

[3] He W, Gan J. The relationship between self-reflection and mental health: a meta-analysis review. Curr Psychol. 2025;44(5):3899–913. doi: 10.1007/s12144-025-07415-9

[4] Krishnamoorthy G, Davis P, O’Donovan A, McDermott B, Mullens A. Through Benevolent Eyes: the Differential Efficacy of Perspective Taking and Cognitive Reappraisal on the Regulation of Shame. Int J Cogn Ther. 2021;14(2):263–88. doi: 10.1007/s41811-020-00085-4 32904830 PMC7462113

[5] Nelson B. Varieties of self-disturbance: a prism through which to view mental disorder. Early Interv Psychiatry. 2013;7(3):231–4. doi: 10.1111/eip.12080 23879831

[6] Philippi CL, Koenigs M. The neuropsychology of self-reflection in psychiatric illness. J Psychiatr Res. 2014;54:55–63. doi: 10.1016/j.jpsychires.2014.03.004 24685311 PMC4022422

[7] Harrison LA, Kats A, Williams ME, Aziz-Zadeh L. The Importance of Sensory Processing in Mental Health: A Proposed Addition to the Research Domain Criteria (RDoC) and Suggestions for RDoC 2.0. Front Psychol. 2019;10:103. doi: 10.3389/fpsyg.2019.00103 30804830 PMC6370662

[8] Dunn W, Griffith JW, Sabata D, Morrison MT, MacDermid JC, Darragh A, et al. Measuring change in somatosensation across the lifespan. Am J Occup Ther. 2015;69(3):6903290020p1-9. doi: 10.5014/ajot.2015.014845 25871603 PMC4453040

[9] Mehling WE, Price C, Daubenmier JJ, Acree M, Bartmess E, Stewart A. The Multidimensional Assessment of Interoceptive Awareness (MAIA). PLoS One. 2012;7(11):e48230. doi: 10.1371/journal.pone.0048230 23133619 PMC3486814

[10] Jones A, Silas J, Todd J, Stewart A, Acree M, Coulson M, et al. Exploring the Multidimensional Assessment of Interoceptive Awareness in youth aged 7-17 years. J Clin Psychol. 2021;77(3):661–82. doi: 10.1002/jclp.23067 33035384

[11] Craig AD. How do you feel? Interoception: the sense of the physiological condition of the body. Nat Rev Neurosci. 2002;3(8):655–66. doi: 10.1038/nrn894 12154366

[12] Aron EN. High Sensitivity as One Source of Fearfulness and Shyness: Preliminary Research and Clinical Implications. In: Extreme fear, shyness, and social phobia: Origins, biological mechanisms, and clinical outcomes. New York, NY: Oxford University Press; 1999. p. 251–72. doi: 10.1093/acprof:oso/9780195118872.003.0014

[13] van den Boogert F, Klein K, Spaan P, Sizoo B, Bouman YHA, Hoogendijk WJG, et al. Sensory processing difficulties in psychiatric disorders: A meta-analysis. J Psychiatr Res. 2022;151:173–80. doi: 10.1016/j.jpsychires.2022.04.020 35489177

[14] Baranek GT, Watson LR, Boyd BA, Poe MD, David FJ, McGuire L. Hyporesponsiveness to social and nonsocial sensory stimuli in children with autism, children with developmental delays, and typically developing children. Dev Psychopathol. 2013;25(2):307–20. doi: 10.1017/S0954579412001071 23627946 PMC3641693

[15] Donnellan AM, Hill DA, Leary MR. Rethinking autism: implications of sensory and movement differences for understanding and support. Front Integr Neurosci. 2013;6:124. doi: 10.3389/fnint.2012.00124 23372546 PMC3556589

[16] Romero-Ayuso D, Maciver D, Richmond J, Jorquera-Cabrera S, Garra-Palud L, Zabala-Baños C, et al. Tactile Discrimination, Praxis and Cognitive Impulsivity in ADHD Children: A Cross-Sectional Study. Int J Environ Res Public Health. 2020;17(6):1897. doi: 10.3390/ijerph17061897 32183331 PMC7143737

[17] Garvey MA, Cuthbert BN. Developing a Motor Systems Domain for the NIMH RDoC Program. Schizophr Bull. 2017;43(5):935–6. doi: 10.1093/schbul/sbx095 28911051 PMC5581884

[18] Brand-Gothelf A, Parush S, Eitan Y, Admoni S, Gur E, Stein D. Sensory modulation disorder symptoms in anorexia nervosa and bulimia nervosa: A pilot study. Int J Eat Disord. 2016;49(1):59–68. doi: 10.1002/eat.22460 26354076

[19] Kitajima T, Otani R, Inoue T, Matsushima N, Matsubara N, Sakuta R. Sensory processing in children and adolescents shortly after the onset of anorexia nervosa: a pilot study. Biopsychosoc Med. 2022;16(1):27. doi: 10.1186/s13030-022-00256-z 36510231 PMC9743604

[20] Saure E, Lepistö-Paisley T, Raevuori A, Laasonen M. Atypical Sensory Processing Is Associated With Lower Body Mass Index and Increased Eating Disturbance in Individuals With Anorexia Nervosa. Front Psychiatry. 2022;13:850594. doi: 10.3389/fpsyt.2022.850594 35432034 PMC9008215

[21] Benarous X, Bury V, Lahaye H, Desrosiers L, Cohen D, Guilé JM. Sensory Processing Difficulties in Youths With Disruptive Mood Dysregulation Disorder. Front Psychiatry. 2020;11:164. doi: 10.3389/fpsyt.2020.00164 32265752 PMC7104792

[22] Cervin M. Sensory Processing Difficulties in Children and Adolescents with Obsessive-Compulsive and Anxiety Disorders. Res Child Adolesc Psychopathol. 2023;51(2):223–32. doi: 10.1007/s10802-022-00962-w 36149521 PMC9867656

[23] Houghton DC, Stein DJ, Cortese BM. Review: Exteroceptive Sensory Abnormalities in Childhood and Adolescent Anxiety and Obsessive-Compulsive Disorder: A Critical Review. J Am Acad Child Adolesc Psychiatry. 2020;59:78–87. doi: 10.1016/j.jaac.2019.06.007 31265873

[24] Tavassoli T, Miller LJ, Schoen SA, Jo Brout J, Sullivan J, Baron-Cohen S. Sensory reactivity, empathizing and systemizing in autism spectrum conditions and sensory processing disorder. Dev Cogn Neurosci. 2018;29:72–7. doi: 10.1016/j.dcn.2017.05.005 28579480 PMC6987900

[25] Tomchek SD, Little LM, Myers J, Dunn W. Sensory Subtypes in Preschool Aged Children with Autism Spectrum Disorder. J Autism Dev Disord. 2018;48(6):2139–47. doi: 10.1007/s10803-018-3468-2 29417432

[26] Garfinkel SN, Seth AK, Barrett AB, Suzuki K, Critchley HD. Knowing your own heart: distinguishing interoceptive accuracy from interoceptive awareness. Biol Psychol. 2015;104:65–74. doi: 10.1016/j.biopsycho.2014.11.004 25451381

[27] Baiano C, Job X, Kirsch LP, Auvray M. Interoceptive abilities facilitate taking another’s spatial perspective. Sci Rep. 2023;13(1):10064. doi: 10.1038/s41598-023-36173-6 37344510 PMC10284897

[28] Schuette SA, Zucker NL, Smoski MJ. Do interoceptive accuracy and interoceptive sensibility predict emotion regulation? Psychol Res. 2021;85(5):1894–908. doi: 10.1007/s00426-020-01369-2 32556535

[29] Vabba A, Porciello G, Monti A, Panasiti MS, Aglioti SM. A longitudinal study of interoception changes in the times of COVID-19: Effects on psychophysiological health and well-being. Heliyon. 2023;9(4):e14951. doi: 10.1016/j.heliyon.2023.e14951 37035351 PMC10065811

[30] Brown TA, Vanzhula IA, Reilly EE, Levinson CA, Berner LA, Krueger A, et al. Body mistrust bridges interoceptive awareness and eating disorder symptoms. J Abnorm Psychol. 2020;129(5):445–56. doi: 10.1037/abn0000516 32202809 PMC8140607

[31] Jenkinson PM, Taylor L, Laws KR. Self-reported interoceptive deficits in eating disorders: A meta-analysis of studies using the eating disorder inventory. J Psychosom Res. 2018;110:38–45. doi: 10.1016/j.jpsychores.2018.04.005 29764604

[32] Pollatos O, Kurz A-L, Albrecht J, Schreder T, Kleemann AM, Schöpf V, et al. Reduced perception of bodily signals in anorexia nervosa. Eat Behav. 2008;9(4):381–8. doi: 10.1016/j.eatbeh.2008.02.001 18928900

[33] Domschke K, Stevens S, Pfleiderer B, Gerlach AL. Interoceptive sensitivity in anxiety and anxiety disorders: an overview and integration of neurobiological findings. Clin Psychol Rev. 2010;30(1):1–11. doi: 10.1016/j.cpr.2009.08.008 19751958

[34] Perkins NM, Ortiz SN, Smith AR, Brausch AM. Suicidal Ideation and Eating Disorder Symptoms in Adolescents: The Role of Interoceptive Deficits. Behav Ther. 2021;52(5):1093–104. doi: 10.1016/j.beth.2021.03.005 34452664 PMC8403232

[35] Calzo JP, Sonneville KR, Haines J, Blood EA, Field AE, Austin SB. The development of associations among body mass index, body dissatisfaction, and weight and shape concern in adolescent boys and girls. J Adolesc Health. 2012;51(5):517–23. doi: 10.1016/j.jadohealth.2012.02.021 23084175 PMC3479441

[36] Craike M, Young JA, Symons CM, Pain MD, Harvey JT, Eime RM, et al. Trends in body image of adolescent females in metropolitan and non-metropolitan regions: a longitudinal study. BMC Public Health. 2016;16(1):1143. doi: 10.1186/s12889-016-3815-1 27825373 PMC5101732

[37] Frisén A, Lunde C, Berg AI. Developmental patterns in body esteem from late childhood to young adulthood: A growth curve analysis. Eur J Dev Psychol. 2014;12(1):99–115. doi: 10.1080/17405629.2014.951033

[38] Steinberg L, Monahan KC. Age differences in resistance to peer influence. Dev Psychol. 2007;43(6):1531–43. doi: 10.1037/0012-1649.43.6.1531 18020830 PMC2779518

[39] Bisi MC, Stagni R. Development of gait motor control: what happens after a sudden increase in height during adolescence? Biomed Eng Online. 2016;15(1):47. doi: 10.1186/s12938-016-0159-0 27197813 PMC4874000

[40] Viel S, Vaugoyeau M, Assaiante C. Adolescence: a transient period of proprioceptive neglect in sensory integration of postural control. Motor Control. 2009;13(1):25–42. doi: 10.1123/mcj.13.1.25 19246776

[41] Murphy J, Brewer R, Catmur C, Bird G. Interoception and psychopathology: A developmental neuroscience perspective. Dev Cogn Neurosci. 2017;23:45–56. doi: 10.1016/j.dcn.2016.12.006 28081519 PMC6987654

[42] Prentice F, Murphy J. Sex differences in interoceptive accuracy: A meta-analysis. Neurosci Biobehav Rev. 2022;132:497–518. doi: 10.1016/j.neubiorev.2021.11.030 34838927

[43] Yang H-X, Hu H-X, Zhang Y-J, Wang Y, Lui SSY, Chan RCK. A network analysis of interoception, self-awareness, empathy, alexithymia, and autistic traits. Eur Arch Psychiatry Clin Neurosci. 2022;272(2):199–209. doi: 10.1007/s00406-021-01274-8 33987711

[44] Howard LM, Ehrlich AM, Gamlen F, Oram S. Gender-neutral mental health research is sex and gender biased. Lancet Psychiatry. 2017;4(1):9–11. doi: 10.1016/S2215-0366(16)30209-7 27856394

[45] Borsboom D, Cramer AOJ. Network analysis: an integrative approach to the structure of psychopathology. Annu Rev Clin Psychol. 2013;9:91–121. doi: 10.1146/annurev-clinpsy-050212-185608 23537483

[46] Borsboom D, Deserno MK, Rhemtulla M, Epskamp S, Fried EI, McNally RJ, et al. Network analysis of multivariate data in psychological science. Nat Rev Methods Primers. 2021;1(1):58. doi: 10.1038/s43586-021-00055-w

[47] Monteleone AM, Cascino G. A systematic review of network analysis studies in eating disorders: Is time to broaden the core psychopathology to non specific symptoms. Eur Eat Disord Rev. 2021;29(4):531–47. doi: 10.1002/erv.2834 33942439 PMC8251923

[48] Golino H, Christensen A, Moulder R, Garrido LE, Jamison L, Shi D. EGAnet: Exploratory Graph Analysis – a Framework for Estimating the Number of Dimensions in Multivariate Data using Network Psychometrics. 2025. Available from: https://cran.r-project.org/web/packages/EGAnet/index.html

[49] Golino HF, Epskamp S. Exploratory graph analysis: A new approach for estimating the number of dimensions in psychological research. Voracek M, editor. PLoS One. 2017;12(6):e0174035. doi: 10.1371/journal.pone.0174035 28594839 PMC5465941

[50] Jones PJ, Ma R, McNally RJ. Bridge Centrality: A Network Approach to Understanding Comorbidity. Multivariate Behav Res. 2021;56(2):353–67. doi: 10.1080/00273171.2019.1614898 31179765

[51] van Borkulo CD, van Bork R, Boschloo L, Kossakowski JJ, Tio P, Schoevers RA, et al. Comparing network structures on three aspects: A permutation test. Psychol Methods. 2023;28(6):1273–85. doi: 10.1037/met0000476 35404628

[52] Petersen AC, Crockett L, Richards M, Boxer A. A self-report measure of pubertal status: Reliability, validity, and initial norms. J Youth Adolesc. 1988;17(2):117–33. doi: 10.1007/BF01537962 24277579

[53] Grimby G, Börjesson M, Jonsdottir IH, Schnohr P, Thelle DS, Saltin B. The “Saltin-Grimby Physical Activity Level Scale” and its application to health research. Scand J Med Sci Sports. 2015;25(Suppl 4):119–25. doi: 10.1111/sms.12611 26589125

[54] Ni X, Shao X, Geng Y, Qu R, Niu G, Wang Y. Development of the Social Media Engagement Scale for Adolescents. Front Psychol. 2020;11:701. doi: 10.3389/fpsyg.2020.00701 32411042 PMC7198835

[55] Brown C, Dunn W. Adolescent/Adult Sensory Profile. San Antonio, TX, USA: Pearson; 2002.

[56] Wilhelm FH, Kochar AS, Roth WT, Gross JJ. Social anxiety and response to touch: incongruence between self-evaluative and physiological reactions. Biol Psychol. 2001;58(3):181–202. doi: 10.1016/s0301-0511(01)00113-2 11698114

[57] Valls M, Rousseau A, Chabrol H. Étude de validation de la version française du Body Esteem Scale (BES) dans la population masculine. J Thérapie Comport Cogn. 2011;21(2):58–64. doi: 10.1016/j.jtcc.2011.02.001

[58] Mendelson BK, Mendelson MJ, White DR. Body-esteem scale for adolescents and adults. J Pers Assess. 2001;76(1):90–106. doi: 10.1207/S15327752JPA7601_6 11206302

[59] Rodgers RF, Schaefer LM, Thompson JK, Girard M, Bertrand M, Chabrol H. Psychometric properties of the Sociocultural Attitudes Towards Appearance Questionnaire-4 (SATAQ-4) in French women and men. Body Image. 2016;17:143–51. doi: 10.1016/j.bodyim.2016.03.002 27081747

[60] Schaefer LM, Burke NL, Thompson JK, Dedrick RF, Heinberg LJ, Calogero RM, et al. Development and validation of the Sociocultural Attitudes Towards Appearance Questionnaire-4 (SATAQ-4). Psychol Assess. 2015;27(1):54–67. doi: 10.1037/a0037917 25285718

[61] Pelletier LG, Vallerand RJ. L’Échelle Révisée de Conscience de Soi: Une traduction et une validation canadienne-française du Revised Self-Consciousness Scale = The Revised Self-Consciousness Scale: A translation and a French-Canadian validation of the Revised Self-Consciousness Scale. Can J Behav Sci Rev Can Sci Comport. 1990;22:191–206. doi: 10.1037/h0078983

[62] Scheier MF, Carver CS. The Self‐Consciousness Scale: A Revised Version for Use with General Populations1. J Applied Social Pyschol. 1985;15(8):687–99. doi: 10.1111/j.1559-1816.1985.tb02268.x

[63] Fenigstein A, Scheier MF, Buss AH. Public and private self-consciousness: Assessment and theory. J Consult Clin Psychol. 1975;43(4):522–7. doi: 10.1037/h0076760

[64] Gilet A-L, Mella N, Studer J, Grühn D, Labouvie-Vief G. Assessing dispositional empathy in adults: A French validation of the Interpersonal Reactivity Index (IRI). Can J Behav Sci Re Can Sci Comport. 2013;45(1):42–8. doi: 10.1037/a0030425

[65] Davis MH. Interpersonal reactivity index. 1980.

[66] Hawk ST, Keijsers L, Branje SJT, der Graaff JV, de Wied M, Meeus W. Examining the Interpersonal Reactivity Index (IRI) among early and late adolescents and their mothers. J Pers Assess. 2013;95(1):96–106. doi: 10.1080/00223891.2012.696080 22731809

[67] Jones P. Networktools: Tools for identifying important nodes in networks. R Package Version 1.2. 0. 2017.

[68] Tomei G, Pieroni MF, Tomba E. Network analysis studies in patients with eating disorders: A systematic review and methodological quality assessment. Int J Eat Disord. 2022;55(12):1641–69. doi: 10.1002/eat.23828 36256543

[69] McNally RJ. Network Analysis of Psychopathology: Controversies and Challenges. Annu Rev Clin Psychol. 2021;17:31–53. doi: 10.1146/annurev-clinpsy-081219-092850 33228401

[70] Epskamp S, Cramer AOJ, Waldorp LJ, Schmittmann VD, Borsboom D. qgraph: Network Visualizations of Relationships in Psychometric Data. J Stat Soft. 2012;48(4). doi: 10.18637/jss.v048.i04

[71] Epskamp S, Borsboom D, Fried EI. Estimating psychological networks and their accuracy: A tutorial paper. Behav Res Methods. 2018;50(1):195–212. doi: 10.3758/s13428-017-0862-1 28342071 PMC5809547

[72] Fried EI, Cramer AOJ. Moving Forward: Challenges and Directions for Psychopathological Network Theory and Methodology. Perspect Psychol Sci. 2017;12(6):999–1020. doi: 10.1177/1745691617705892 28873325

[73] Isvoranu A-M, Epskamp S. Which estimation method to choose in network psychometrics? Deriving guidelines for applied researchers. Psychol Methods. 2023;28(4):925–46. doi: 10.1037/met0000439 34843277

[74] Williams DR, Rast P. Back to the basics: Rethinking partial correlation network methodology. Br J Math Stat Psychol. 2020;73(2):187–212. doi: 10.1111/bmsp.12173 31206621 PMC8572131

[75] Bringmann LF, Elmer T, Epskamp S, Krause RW, Schoch D, Wichers M, et al. What do centrality measures measure in psychological networks?. J Abnorm Psychol. 2019;128(8):892–903. doi: 10.1037/abn0000446 31318245

[76] Neubeck M, Johann VE, Karbach J, Könen T. Age-differences in network models of self-regulation and executive control functions. Dev Sci. 2022;25(5):e13276. doi: 10.1111/desc.13276 35535463

[77] Lapp HS, Croy I. Insights from the German Version of the Social Touch Questionnaire: How Attitude towards Social Touch relates to Symptoms of Social Anxiety. Neuroscience. 2021;464:133–42. doi: 10.1016/j.neuroscience.2020.07.012 32673628

[78] Cascio CJ, Moore D, McGlone F. Social touch and human development. Dev Cogn Neurosci. 2019;35:5–11. doi: 10.1016/j.dcn.2018.04.009 29731417 PMC6968965

[79] Ma Y-K, Zeng P-Y, Chu Y-H, Lee C-L, Cheng C-C, Chen C-H, et al. Lack of social touch alters anxiety-like and social behaviors in male mice. Stress. 2022;25(1):134–44. doi: 10.1080/10253890.2022.2047174 35254226

[80] May AC, Stewart JL, Tapert SF, Paulus MP. The effect of age on neural processing of pleasant soft touch stimuli. Front Behav Neurosci. 2014;8:52. doi: 10.3389/fnbeh.2014.00052 24600366 PMC3930859

[81] Seth AK. Interoceptive inference, emotion, and the embodied self. Trends Cogn Sci. 2013;17(11):565–73. doi: 10.1016/j.tics.2013.09.007 24126130

[82] Crone EA, Fuligni AJ. Self and Others in Adolescence. Annu Rev Psychol. 2020;71:447–69. doi: 10.1146/annurev-psych-010419-050937 31337274

[83] Todd J, Aspell JE, Barron D, Swami V. An exploration of the associations between facets of interoceptive awareness and body image in adolescents. Body Image. 2019;31:171–80. doi: 10.1016/j.bodyim.2019.10.004 31654981

[84] Juarascio AS, Perone J, Timko CA. Moderators of the relationship between body image dissatisfaction and disordered eating. Eat Disord. 2011;19(4):346–54. doi: 10.1080/10640266.2011.584811 22352974

[85] Castro D, Ferreira F, de Castro I, Rodrigues AR, Correia M, Ribeiro J, et al. The Differential Role of Central and Bridge Symptoms in Deactivating Psychopathological Networks. Front Psychol. 2019;10:2448. doi: 10.3389/fpsyg.2019.02448 31827450 PMC6849493

[86] Mehling WE, Acree M, Stewart A, Silas J, Jones A. The Multidimensional Assessment of Interoceptive Awareness, Version 2 (MAIA-2). Costantini M, editor. PLoS One. 2018;13(12):e0208034. doi: 10.1371/journal.pone.0208034 30513087 PMC6279042

[87] Halliwell E, Jarman H, Tylka T, Slater A. Adapting the Body Appreciation Scale-2 for Children: A psychometric analysis of the BAS-2C. Body Image. 2017;21:97–102. doi: 10.1016/j.bodyim.2017.03.005 28414994

[88] Boltanski L. Les usages sociaux du corps. Ann Hist Sci Soc. 1971;26(1):205–33. doi: 10.3406/ahess.1971.422470

