# Supplementary material for: Psychometric network analysis reveals how sensory processing relates to self-reflection traits in adolescence
Source: PLoS One. 2025 Dec 1;20(12):e0335259. doi: 10.1371/journal.pone.0335259 (PMC12668497; doi:10.1371/journal.pone.0335259)
Supplement: S1 File — This document contains additional information regarding the psychometric scales used, data preprocessing, detailed results using alternative estimation methods, robustness analyses — namely edge weight estimates, differences between edges, and case-dropping bootstrap —, as well as additional descriptive statistics for subgroups. (DOCX) [file pone.0335259.s001.docx]

Psychometric network analysis reveals how sensory processing relates to self-reflection traits in adolescence

# 1 Supplementary Information regarding method

## 1.1 Sample recruitment

We conducted a voluntary and anonymous online survey accessible on Limesurvey (LimeSurvey.org, 2009) between May 2021 and May 2023. Participants were informed that the aim of the study was to better understand “the links between body and mind during adolescence” through a video and an explanatory text presented on the first page of the questionnaire. We collaborated with schools and sports clubs in various regions of France, and launched a campaign through email, social media, and word of mouth, inviting individuals between the ages of 10 and 25 to participate.

## 1.2 Measures

All scales that required answers on a Likert scale were presented as a 5-point scale to facilitate the completion of the survey. All questions were forced-choice, but participants had the option to answer “I do not wish to answer” or “I do not understand the question”. They were also invited to leave comments at the end of the questionnaire. We gathered information about how people joined the study and their zip codes. We also collected data about their dominant hand, native language, school grade, and, for university students, their major. In total, participants answered 163 items, from which we obtained 32 measures for analysis.

### 1.2.1 Puberty stage compared to average

The Pubertal Development Scale (PDS) (Petersen *et al.*, 1988) contains five items assessing pubertal status, including, for both boys and girls, the presence of a growth spurt, pubic hair, and skin changes. For girls, two items also assess breast development and menarche, and for boys, facial hair growth and voice change. There is a significant correlation between PDS and pubertal status estimate through physical exam (Shirtcliff *et al.*, 2009). Each item ranges from 1 (development has not yet begun) to 4 (development seems completed). From the score on the five items, we obtained a continuous puberty index. From the whole sample, we defined the function of the puberty index relative to age by fitting a polynomial model with terms up to the third degree for the variable age and including the variable sex as a predictor (see figure S1). Then for each individual, we calculated the deviation from the curve. This gave us a quantitative index of pubertal development relative to people of the same age. Participants with an absolute value of puberty index compared to mean superior to 3.5 x SD were considered outliers and the response were therefore excluded from the analysis (3 subjects).


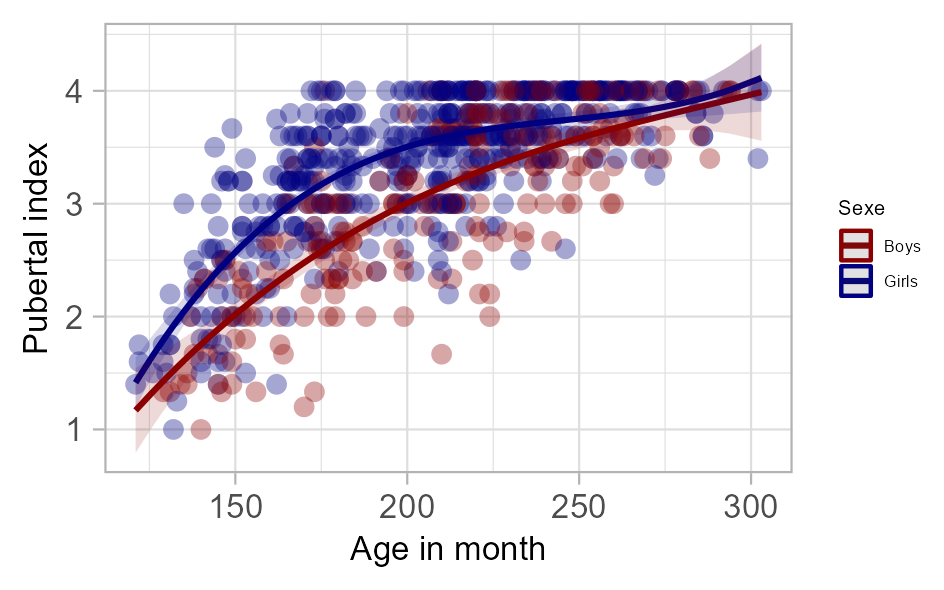


**Figure S1** Puberty indexes according to age for boys and girls

### 1.2.2 Physical activity

We used the Saltin-Grimby Physical Activity Level Scale (SGPA) which is a four-level questionnaire to assess leisure time physical activity (Grimby *et al.*, 2015) and is a valid measure of physical activity levels in adolescents (Beldo *et al.*, 2022). We used the French version recently used by Isoard-Gautheur *et al.* (2023).

### 1.2.3 Affective, behavioral and cognitive engagement in Social Media

We used the 11-items Social Media Engagement Scale For Adolescents (Ni *et al.*, 2020). The questionnaire is made of three subscales: Behavioral engagement included 4 items like “Using social media is my daily habit”, Cognitive engagement has 3 items like “The support and encouragement of others on social media is very important to me”), and Affective engagement consist of 4 items like “I feel bored when I can’t use social media”. The Likert scales ranged from “strongly disagree” (1) to “strongly agree” (5). We translated the questionnaire as no version existed in French. We found good internal consistency (before imputation for missing data, see below) in the whole scale ($\alpha$ = 0.88) and in the three subscales (Behavioral $\alpha$ = 0.9, Cognitive $\alpha$ = 0.74, Affective $\alpha$ = 0.76).

### 1.2.4 Body Noticing, not-distracting, not-worrying, listening and trusting and emotional awareness of the body: six subscales of Multidimensional Assessment of Interoceptive Awareness (MAIA)

Noticing: 4 items like “I notice changes in my breathing such as whether it speeds up or slows down”. Not-Distracting: 3 items like “I distract myself from sensations of discomfort”. Not-Worrying: 3 items like “ I start to worry that something is wrong if I feel any discomfort”. Emotional Awareness: 5 items like ““I notice how my body changes when I feel happy/joyful”. Body Listening: 3 items like “ When I am upset, I take time to explore how my body feels”. Body Trusting: 3 items like “I trust my body sensations”. Internal consistency across all items in the present study was $\alpha$ = 0.76. (Noticing $\alpha$ = 0.67, Not-Distracting $\alpha$ = 0.65, Not-Worrying $\alpha$ = 0.55, Emotional Awareness $\alpha$ = 0.79, Body Listening $\alpha$ = 0.77, Body Trusting $\alpha$ = 0.79).

### 1.2.5 Sensitivity Threshold and Behavior to Sensory Stimulation

Sensory threshold: 14 items like “I don’t feel comfortable wearing certain fabrics (for example wool, silk, velvet, clothing labels…)”. Behavioral response to sensation: 9 items like “I avoid elevators and/or escalators because I don’t like movement”. In the present study, internal consistency across items related to sensitivity threshold was $\alpha$ = 0.68 and, across those related to behavioral response, was $\alpha$ = 0.44.

### 1.2.6 Social Touch

Originally, the questionnaire was composed of 8-items. 5 items like “I would rather avoid shaking hands” originated from Social Touch Questionnaire (Wilhelm *et al.*, 2001) and 3 items like “I generally seek physical contact with others” originated from Dunn Sensory Profile (Brown et Dunn, 2002). One item (“I like petting animals”) was dropped from the analysis as it was significantly not consistent with other items. Internal consistency across the seven items in the present study was $\alpha$ = 0.73.

### 1.2.7 Private Self Consciousness, Public Self Consciousness and Social Anxiety

Private Self-Consciousness subscale: 9 items, like “I’m always trying to figure myself out”. Two items (6 and 8) were rephrased to be more easily understandable by younger participants. Public Self-Consciousness subscale: 7 items like “I usually worry about making a good impression”. Social Anxiety subscale: 6 items, like “I have trouble working when someone is watching me”. Two Items (16 and 20) were modified so as not to be appearance-oriented. Internal consistency across all items in the present study was $\alpha$ = 0.86. (Private SCS $\alpha$ = 0.78, Public SCS $\alpha$ = 0.84, Social Anxiety $\alpha$ = 0.79).

### 1.2.8 Perspective Taking and Empathic Concern

Perspective Taking: 7 items like “I try to look at everybody’s side of a disagreement before I make a decision”. Empathic Concern: 7 items like “When I see someone being taken advantage of, I feel kind of protective towards them”. Internal consistency across all items in the present study was $\alpha$ = 0.79 (Perspective Taking $\alpha$ = 0.65, Empathic Concern $\alpha$ = 0.78).

### 1.2.9 Body-Esteem for Appearance, for Weight and Attribution

Appearance: 10 items like “I like what I see when I look in the mirror”. Weight satisfaction: 8 items like “I am satisfied with my weight”. Attribution: 5 items like “People like my looks”. Internal consistency across all items in our sample was $\alpha$ = 0.92 (Appearance $\alpha$ = 0.89, Weight $\alpha$ = 0.92, Attribution $\alpha$ = 0.55).

### 1.2.10 Family, peers and social-media appearance-related pressure

Family subscale: 4 items like “I feel pressure from family members to improve my appearance”. Peers subscale: 4 items like “I feel pressure from my peers to look in better shape”. Social Media subscale: 4 items like “I feel pressure from the media to look in better shape”. Internal consistency across all items in the present study was $\alpha$ = 0.78 (Family $\alpha$ = 0.75, Peers $\alpha$ = 0.62, Attribution $\alpha$ = 0.7).

### 1.2.11 Resistance to Peer Influence

We used the original version. Internal consistency across all items in the present study was $\alpha$ = 0.68.

## 1.3 Missing Data handling

In the analyzed sample, responses to 3.82% of the items were missing. When a single value was missing in a multi-items measure, the score for this measure was computed using the mean of all the answered items. After this among the individual scores for the 32 measures 1.11% was missing. Four measures were missing in more than one percent of the participants: Physical activity (3.7%), number of chronic pain (13.6%), economic status (2.9%) and resistance to peer influence (14.6%). In the first three, this was mostly due to misunderstanding the questions. The resistance to peer influence missing rate was mostly related to mistakes in the completion procedure (answering in two rather than only one group of possible responses). To allow for the largest possible sample size and network comparison tests, we imputed missing measures as follows. Little’s MCAR test was statistically significant (X²(689) = 831.18, p < 0.001), indicating data was not missing at random and could therefore be handled using multivariate imputation (Takahashi, 2017). We used multivariate imputation by chained equations using the *Mice* package in R (Buuren et Groothuis-Oudshoorn, 2011). To check that imputation did not affect the results, we correlated the interrelations of the data-imputed network with the interrelations of the missing-data network, a procedure used by Fritz *et al.* (2019). A high correlation would indicate that results are similar for both data sets and thus that the imputation procedure would not influence the results. The correlation between the data-imputed network and the missing-data network was r = 0.99, indicating that the imputation procedure should not affect the results.

# 2 Supplementary results

## 2.1 Zero-order and partial correlation matrix

Networks constructed using correlations conditioned on numerous variables can lead to false detection of edges (McNally, 2021; Borsboom, 2021; Forbes, 2019; De Ron, 2019; Epskamp & Fried, 2018). To examine the impact of using partial correlations rather than zero-order correlations, and to identify edges that appear only in the partial correlation matrix, potentially due to collider bias or spurious associations, we compared the zero-order (Figure S2) and partial correlation matrices (Figure S3). Specifically, we subtracted the partial correlation matrix from the zero-order correlation matrix. The resulting visualization (Figure S4) highlights the differences between the two: positive values (blue) indicate stronger associations in the zero-order correlation matrix, while negative values (red) reflect stronger associations in the partial correlation matrix, suggesting potentially spurious or adjusted edges. The most prominent differences were observed for variables related to sex and gender. These findings underscore the importance of accounting for sex and gender in future network-based research. Notably, the few edges linking sensory and self-reflection nodes—whose importance increased after adjusting for other variables, as indicated by red coloring—were not retained in the final network estimated using the EBICglasso procedure.


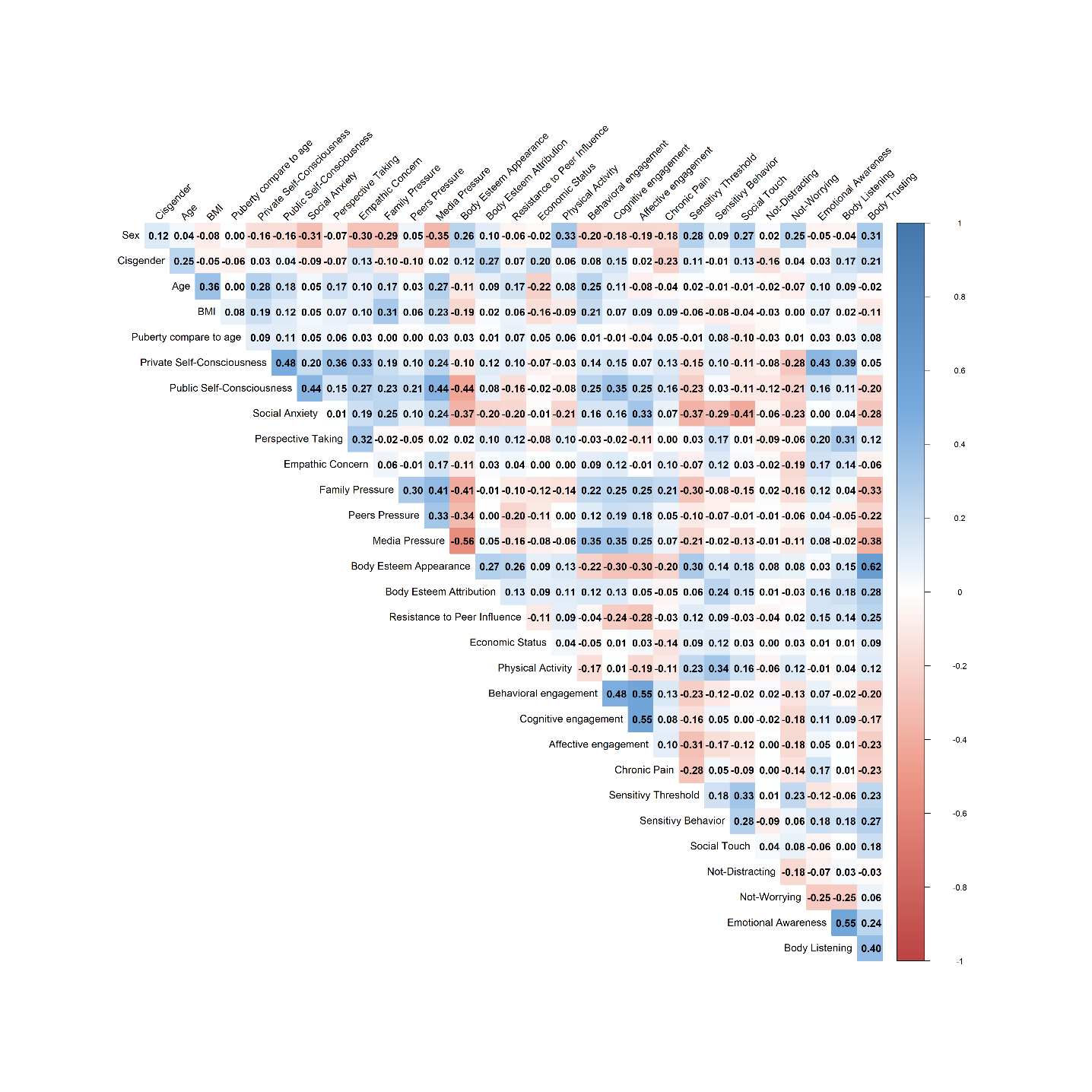


### *Figure S2. Zero-order correlation matrix displaying all pairwise correlations among the variables included as nodes in the network*


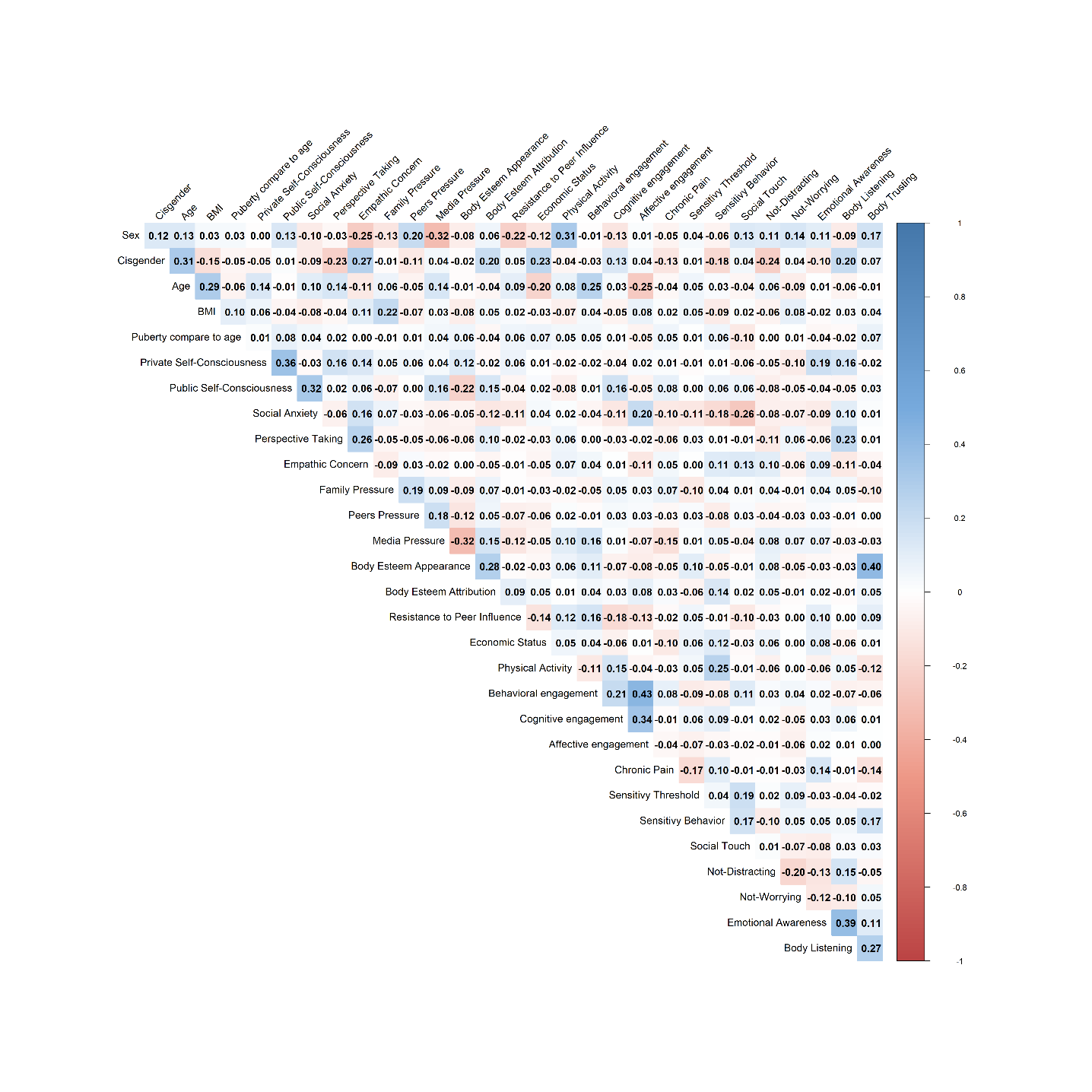


### *Figure S3. Partial correlation matrix of the network variables prior to LASSO regularization, illustrating the unique associations between variables while controlling for all others.*


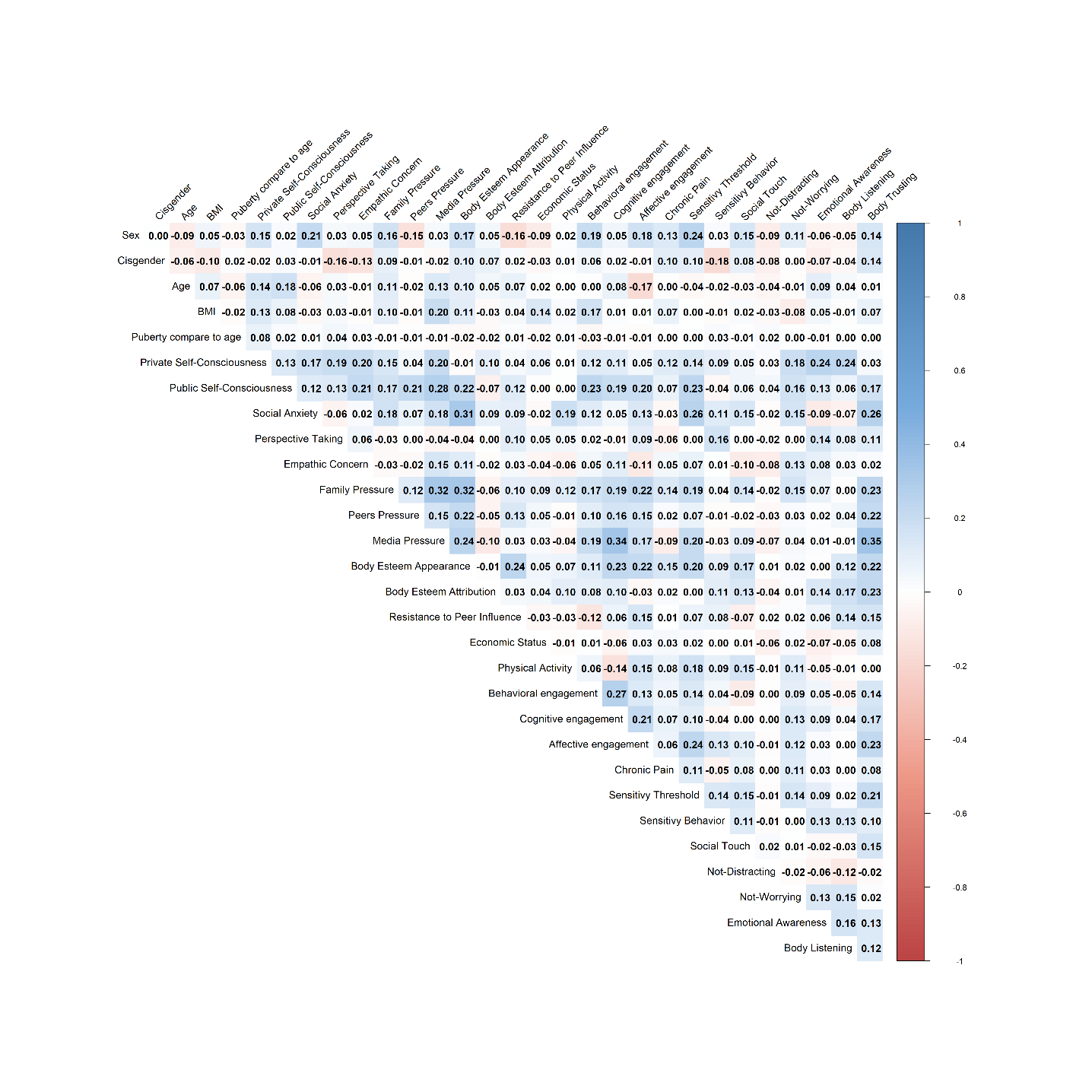


***Figure S4.*** *Comparison matrix contrasting the partial correlation network with the original zero-order correlation matrix. The values represent the raw differences in correlation coefficients, obtained by subtracting the partial correlations from the zero-order correlations. Blue squares indicate cases where the zero-order correlation is stronger than the partial correlation (i.e., a decrease in association strength after adjusting for other variables). Red squares indicate cases where the partial correlation is stronger (i.e., an increase after adjustment). The numbers inside the squares represent the magnitude of the difference, with larger absolute values reflecting greater changes between the two types of correlation.*

## 2.2 Results with other models

We replicated the main analyses using three alternative network estimation methods: a thresholded version of EBICglasso, the ggmModSelect algorithm (Isvoranu & Epskamp, 2023), and unregularized partial correlations with False Discovery Rate (FDR) correction (Williams & Rast, 2020). Although the exact network structures varied slightly, the strongest edges, overall community patterns, and bridge nodes remained largely consistent across models.

However, strength centrality rankings differed substantially across methods. This aligns with prior findings and highlights the need to interpret strength centrality with caution. These discrepancies underscore the importance of focusing on more robust indices, such as bridge centrality, when the goal is to identify variables that connect distinct psychological domains.

In addition, notable sources of variation concerned group differences (e.g., by sex and age). Despite these variations, a consistent pattern emerged regarding sex differences: body trusting played a more prominent role in females, supporting the results discussed in the main manuscript. However, the low stability of age-related differences among girls suggests that these findings should be interpreted with caution, as also emphasized in the discussion section of the main text.

### 2.2.1 Strength centrality


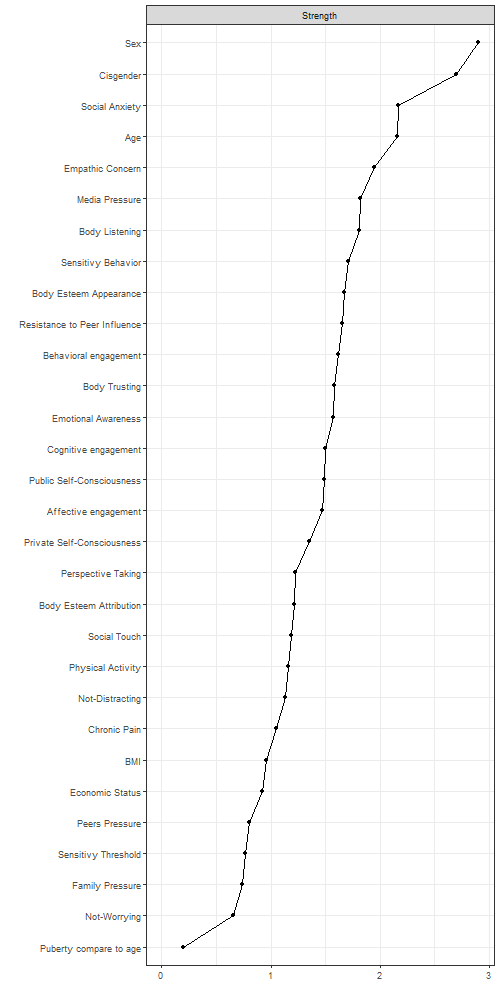

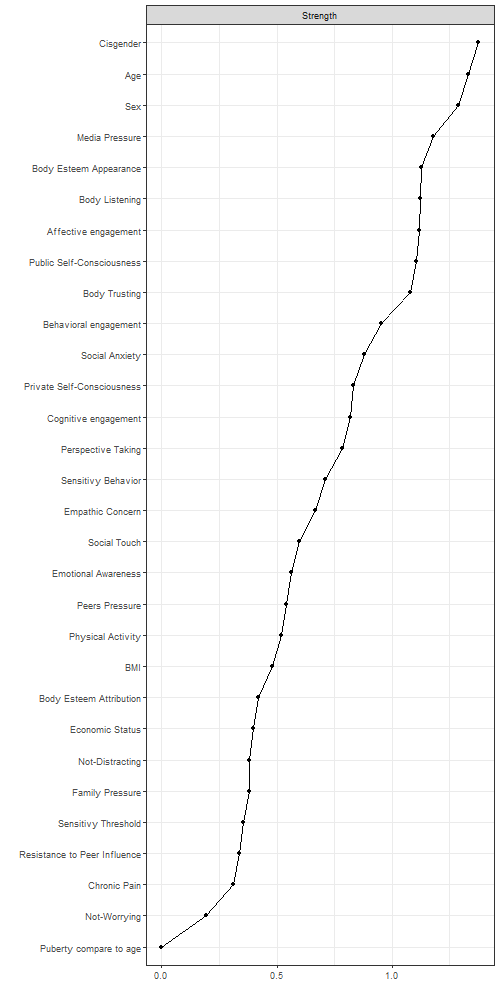

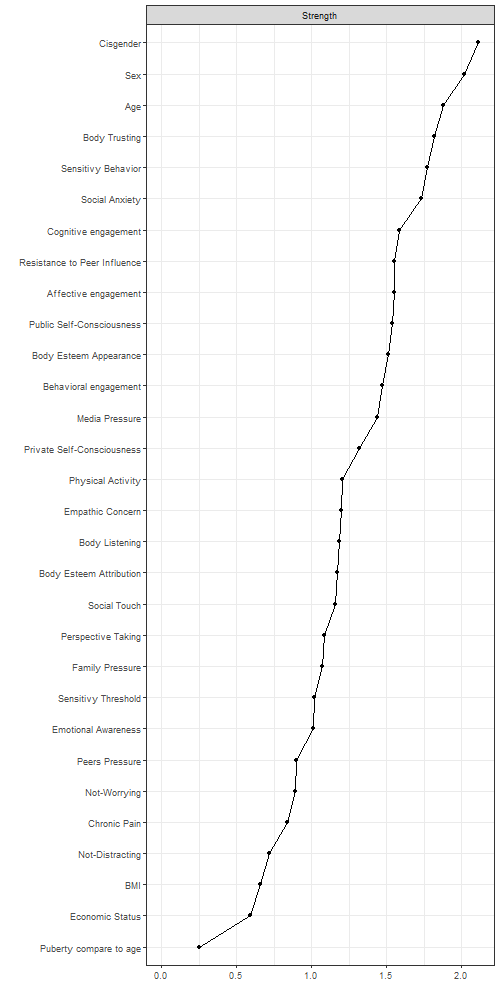


***Figure S5.*** *Strength centrality indices for sensory-processing characteristics and self-reflection traits* *in the network estimated using (a) the EBICglasso method with global threshold (promoting higher specificity), (b) the ggmModSelect method, and (c) partial correlations corrected for multiple comparisons using FDR.*

### 2.2.2 Bridge strength


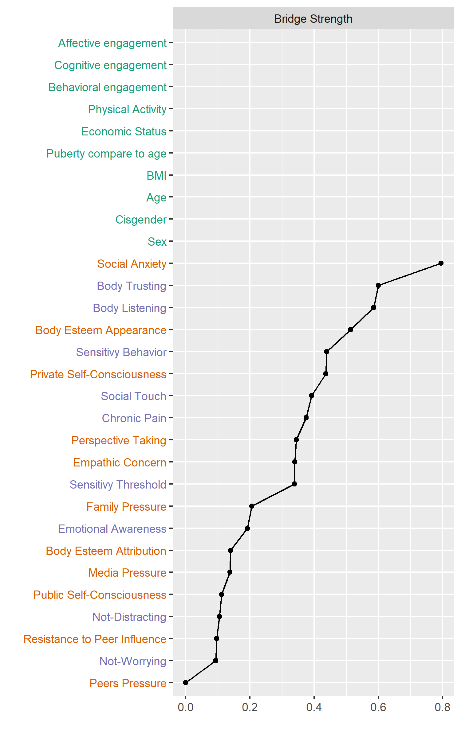

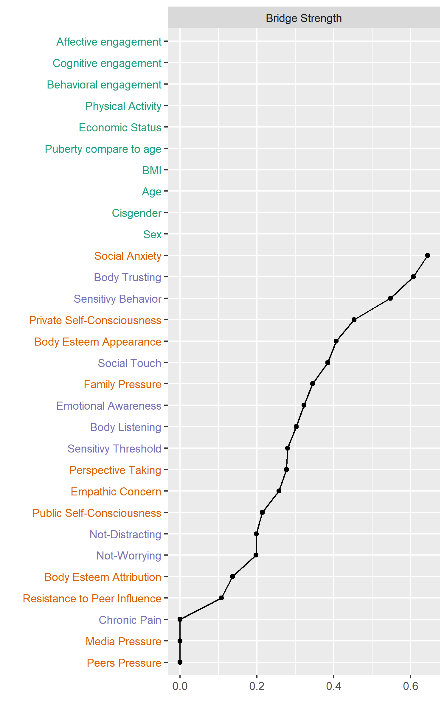

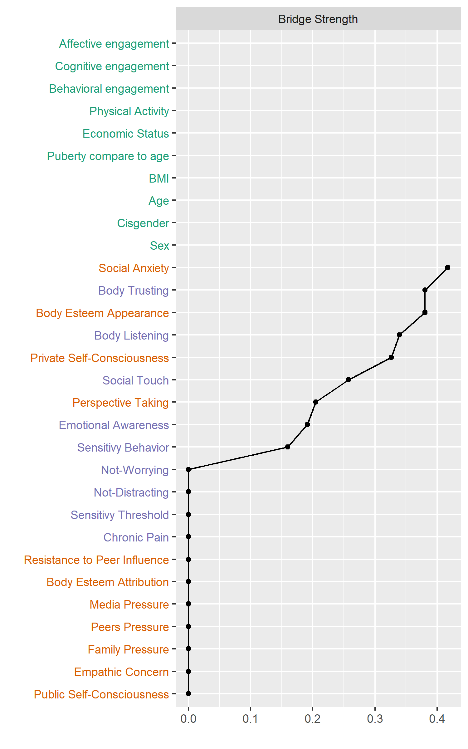


***Figure S6*** *Bridge strength indices for sensory-processing characteristics (purple nodes) and self-reflection traits (orange nodes) in the network estimated using (a) the EBICglasso method with global threshold (promoting higher specificity), and (b) the ggmModSelect method, and (c) partial correlations corrected for multiple comparisons using FDR.*

### 2.2.2 Communities detection


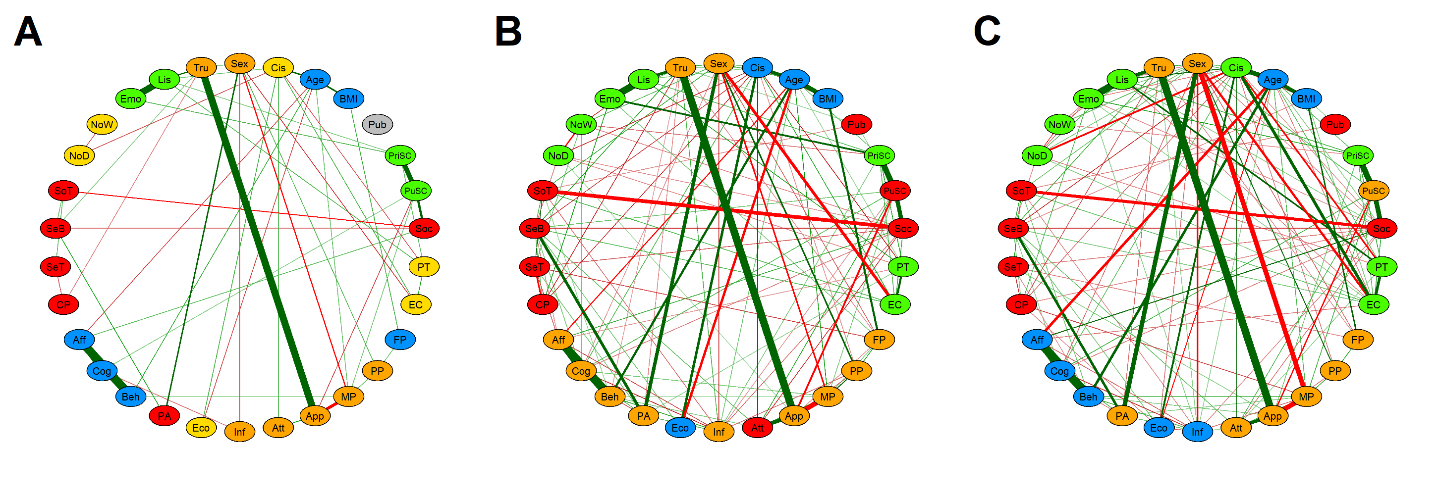


***Figure S7*** *Communities identified by the community.detection function of the exploratory graph analysis applied to network estimated using different models: (a) the EBICglasso method with global threshold (promoting higher specificity), (b) the ggmModSelect method, and (c) partial correlations corrected for multiple comparisons using FDR.*

Despite some variations, all network estimation methods revealed three main communities linking sensory and self-reflection nodes. Community 1 (red) included social anxiety, sensory threshold, sensory behavior, social touch, and physical activity (for the EBICglasso model), indicating a community centered on sensory sensitivity and social anxiety. Community 2 (orange) comprised body trusting and several variables related to body appearance, including body esteem (appearance and—in three out of four models—attribution) and perceived appearance-related pressure from media, peers, and family. Community 3 (green) encompassed private self-consciousness, the two subscales of the Interpersonal Reactivity Index (perspective taking and empathic concern, in three out of four models), as well as variables linked to interoceptive awareness, including emotional awareness, body listening, and—in three out of four models—not-distracting and not-worrying. These patterns suggest a reasonably consistent community structure across methods, broadly distinguishing between three main communities: (1) somatosensation with social anxiety, (2) body trusting with body appearance, and (3) interoception with private self-consciousness and others’ perspectives.

### 2.2.3 Networks comparisons

#### 2.2.3.1 Comparison between sexes

**With EBICglasso with global threshold**
the network invariance test was significant (test statistic M: 0.39, p = 0.022). Global strength comparison tests showed that the difference between the girls’ network global strength (3.60) and the boys’ network global strength (1.20) was not significant (test statistic S: 2.40, p = 0.16).

When not correcting for multiple comparisons, one edge was found to differ significantly between boys and girls. The positive connection between body esteem appearance and body trusting (p = 0.038) was stronger in girls.

No difference was found regarding bridge strength.

Body trusting (p = 0.002), body esteem appearance (p=0.003) and media pressure (p=0.041) showed higher centrality in girls.

However, after applying the Bonferroni-Holm correction, no statistically significant differences remained in edge strength, centrality strength, or bridge strength.

**With ggmModSelect**
The network invariance test was not significant (Test statistic M: 0.27, p = 0.55). Global strength comparison tests showed that the difference between the girls’ network global strength (4.60) and the boys’ network global strength (3.73) was also not significant (Test statistic S: 0.875, p = 0.39). Since the overall level of connectivity was not significantly different between the two groups, differences in individual edge weights are more likely to reflect content-related variations.

When not correcting for multiple comparisons, three edges were found to differ significantly between boys and girls. The negative association between chronic pain and body esteem appearance was stronger in boys (p = 0.005). The positive association between sensitivity behavior and private self-consciousness was stronger in boys (p = 0.015), as was the association between sensitivity behavior and body esteem attribution (p = 0.012).

Body trusting displayed higher bridge strength in girls compared to boys (p = 0.016).

Regarding strength centrality, resistance to peer influence (p = 0.005), and sensitivity threshold (p=0.045) showed higher strength centrality in girls.

After applying the Bonferroni-Holm correction, no statistically significant differences remained in edge strength, strength centrality, or bridge strength.

**With partial correlation model (FDR corrected)**
The network invariance test was not significant (M = 0.29, p = 0.67). Global strength comparison test showed that the difference between the girls’ network (4.22) and the boys’ network (2.02) was also not significant (S = 2.21, p = 0.10). Thus, the overall level of connectivity did not differ significantly between the two groups, suggesting that any differences in individual edge weights are more likely to reflect content-specific variations.

Without correction for multiple comparisons, eight edges linking sensory nodes to self-reflection traits differed significantly between boys and girls. Girls showed stronger positive associations between body trusting and body esteem appearance (p = 0.012) and between body trusting and resistance to peer influence (p = 0.031). In boys, negative associations between chronic pain and social anxiety (p = 0.027), chronic pain and resistance to peer influence (p = 0.008), chronic pain and body esteem appearance (p = 0.012), not-distracting and social anxiety (p = 0.005), and not-distracting and resistance to peer influence (p = 0.023) were stronger. Boys also showed a stronger positive association between body listening and family pressure (p = 0.013).

At the node level, body trusting (p = 0.003) displayed significantly higher bridge strength in girls, while chronic pain (p = 0.002), not-distracting (p = 0.004), and social anxiety (p = 0.046) showed higher bridge strength in boys. Regarding strength centrality, sensitivity threshold (p = 0.008), behavioral engagement (p = 0.038), and body trusting (p = 0.003) were higher in girls, whereas not-distracting (p = 0.003) was higher in boys.

After applying the Bonferroni-Holm correction, no significant differences remained for edge strength. However,

body trusting (p = 0.040) showed significantly higher bridge strength in girls, whereas chronic pain (p = 0.040) and not-distracting (p = 0.041) showed higher bridge strength in boys. Higher strength centrality for body trusting (p = 0.040) in girls and for not-distracting (p = 0.040) in boys also remained statistically significant.

#### 2.2.3.2 Comparison between age groups in girls

**With EBICglasso with global threshold**
The network invariance test was not significant (Test statistic M: 0.36, p = 0.90). Global strength comparison tests showed that the difference between young girls’ global strength (3.07) and older girls’ global strength (1.77) was not significant (Test statistic S: 1.29, p = 0.27). Since the overall level of connectivity was not significantly different between the two groups, differences in individual edge weights are more likely to reflect content-related variations.

When not correcting for multiple comparisons, four edges were found to be significantly different between the two groups: the positive associations between body listening and perspective taking (p = 0.012), between body trusting and body esteem for appearance (p = 0.007) were stronger in younger girls. The negative association

between sensitivity behavior and body esteem for appearance (p < 0.001) was stronger in younger girls. The positive association between body trusting and body esteem attribution was stronger in older girls (p = 0.007)

Four nodes differed in bridge centrality: perspective taking (p = 0.012), body esteem appearance (p < 0.001), and body listening (p = 0.033) displayed higher bridge centrality in younger girls, while body esteem attribution had higher bridge centrality in the older group (p = 0.007).

No difference was found regarding strength centrality.

After correcting for multiple comparisons, the negative association between sensitivity behavior and body esteem appearance (p = 0.049) remained stronger in younger girls, and body esteem appearance (p = 0.047) showed higher bridge centrality in younger girls.

**With** **ggmModSelect**
The network invariance test was not significant (Test statistic M: 0.29, p = 0.50). However, global strength comparison tests revealed a significant difference between young girls’ global strength (5.84) and older girls’ global strength (3.02) (Test statistic S: 2.82, p = 0.007). Since the overall level of connectivity was significantly different between the two groups, differences in individual edge weights may be less reflective of specific content and more influenced by overall network density.

When not correcting for multiple comparisons, four edges were significantly different between the two groups: a positive association between sensitivity behavior and resistance to peer influence was stronger in younger girls (p = 0.017), as were the association between body listening and perspective taking (p = 0.021) and between body listening and media pressure (p = 0.017). The positive association between sensitivity behavior and media pressure was stronger in older girls (p = 0.0036).

No significant differences were found in bridge centrality.

Age showed significantly higher strength centrality in youth (p =0.002), while not worrying (p = 0.040) showed higher strength centrality in the older group.

After applying the Bonferroni-Holm correction, no statistically significant differences remained in edge strength, strength centrality, or bridge strength.

**With partial correlation model (FDR corrected)**
The network invariance test was not significant (M = 0.29, p = 0.90). Global strength comparison test showed that the difference between young girls’ global strength (4.40) and older girls’ global strength (1.97) was also not significant (S = 2.42, p = 0.071).

Without correction for multiple comparisons, five edges differed significantly between the groups. In younger girls, associations were stronger between sensitivity behavior and empathic concern (p = 0.027), sensitivity behavior and body esteem appearance (p = 0.001), body listening and perspective taking (p = 0.030), and body trusting and body esteem appearance (p = 0.004). In contrast, the positive association between body trusting and body esteem attribution (p = 0.004) was stronger in older girls.

For bridge centrality, body esteem appearance (p = 0.012) and body listening (p = 0.034) showed higher values in youth. For strength centrality, body esteem appearance (p = 0.005) and body listening (p = 0.044) were also higher in younger girls. After correction for multiple comparisons, no significant differences remained for edge strength, bridge centrality, or strength centrality.

## 2.3 Robustness analyses on the main model (EBICglasso with gamma = 0.5 without threshold)

### 2.2.1 Accuracy and stability of networks estimates

The accuracy of edge-estimates was assessed using a nonparametric bootstrap procedure implemented using the R-package *Bootnet* (Epskamp *et al.*, 2018 version 1.5.1). To test the accuracy of individual edge weight estimates, their 95 % confidence intervals (CI) were computed using 2000 bootstrap samples (see panel a in figures S4, S5, S6, S7, S8). We checked pairwise differences between edge weights. Computing the difference of the bootstrap values we construct a CI for edge weight differences, and we checked if it contained 0 (see panel b in figures S4, S5, S6, S7, S8).

We evaluated the robustness of strength centrality and bridge centrality by employing the subsamples bootstrap method implemented in the *bootnet* package (2000 subsamples).

In the whole sample, for strength centrality coefficient stability (CS) was CS=0.72, regarding bridge-strength centrality CS=0.69.

Centrality indexes for subgroups were not investigated as CS were not particularly high.

Strength centrality: CS=0.57 in girls, CS=0.2 in boys, CS=0.4 in younger girls, and CS=0.37 in older girls.

Bridge strength centrality: CS =0.69 in girls, CS=0.2 in boys, CS=0.46 in younger girls, and CS=0.43 in older girls.


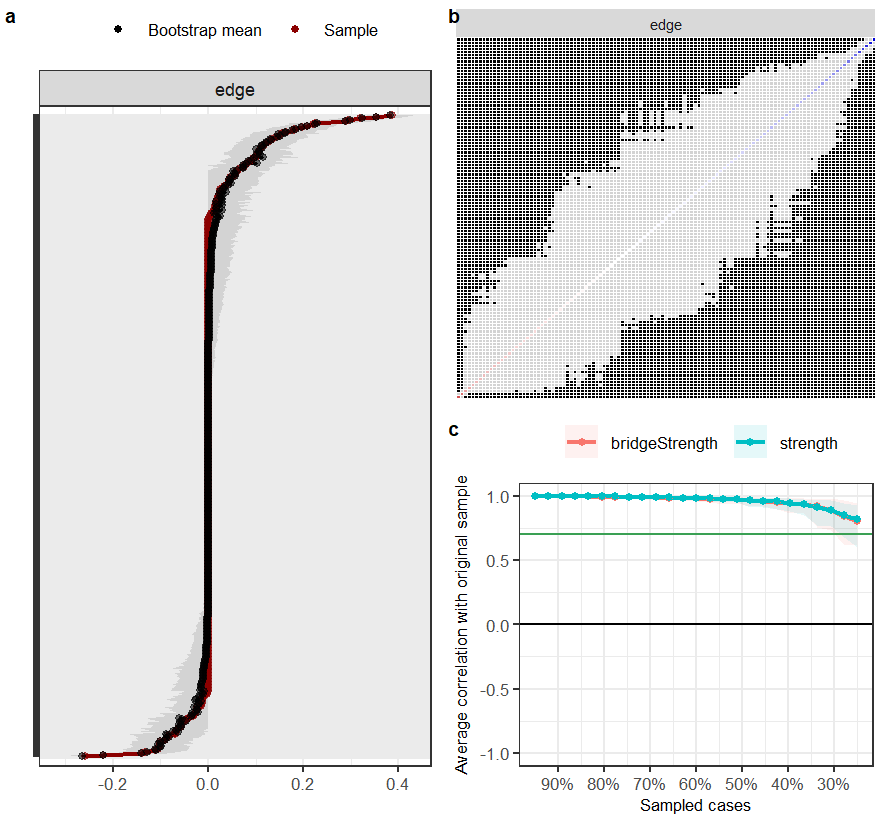


**Figure S8** Robustness analyses. a) Edge weight estimates. The sample value (red line), bootstrapped 95 % intervals (shaded area) and average bootstrapped value (black line) of edge weights in the whole sample. b) Differences between edges. Each square corresponds to an edge couple, the black squares correspond to edges that where significantly different (whose CI were not overlapping). c) Case-dropping bootstrap analysis showing average correlation between strength indexes (strength centrality and bridge-strength) estimated in the whole sample and strength indexes (strength centrality and bridge-strength) estimated on a random subsample, retaining only a certain portion of cases.


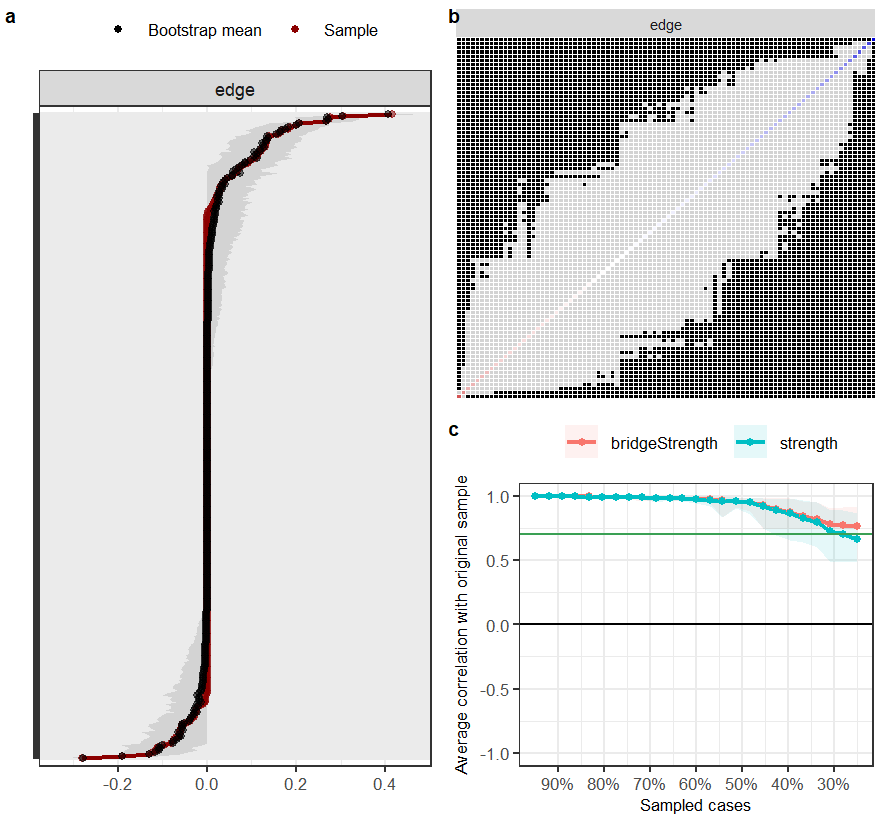


**Figure S9** As in figure S4 for the girls group


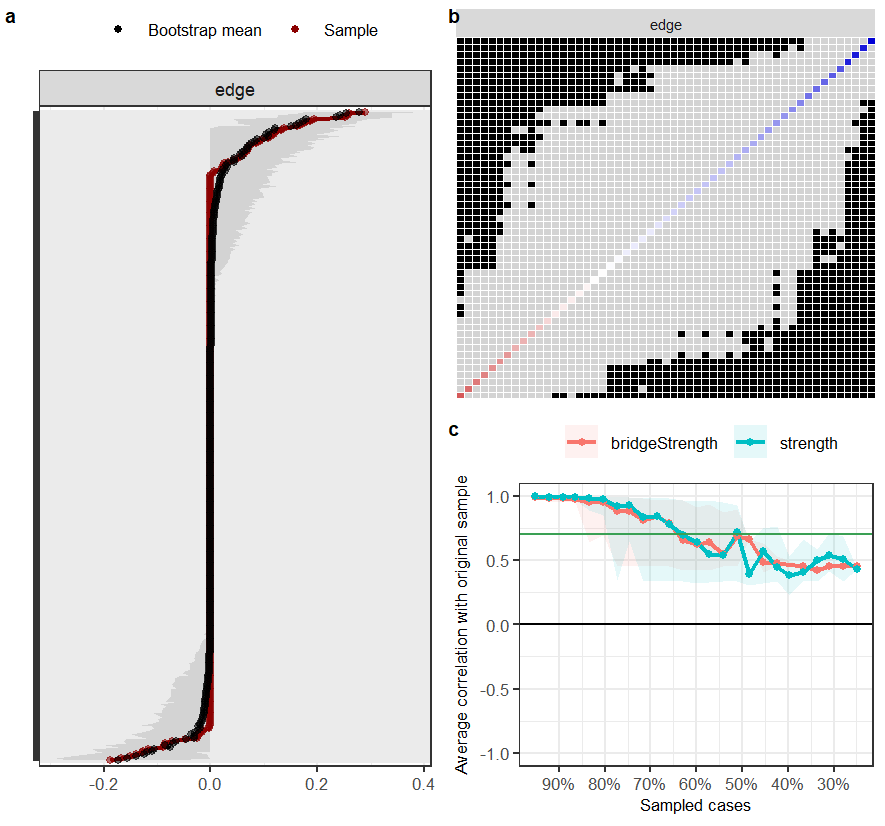


**Figure S10** As in figure S4 for the boys’ group


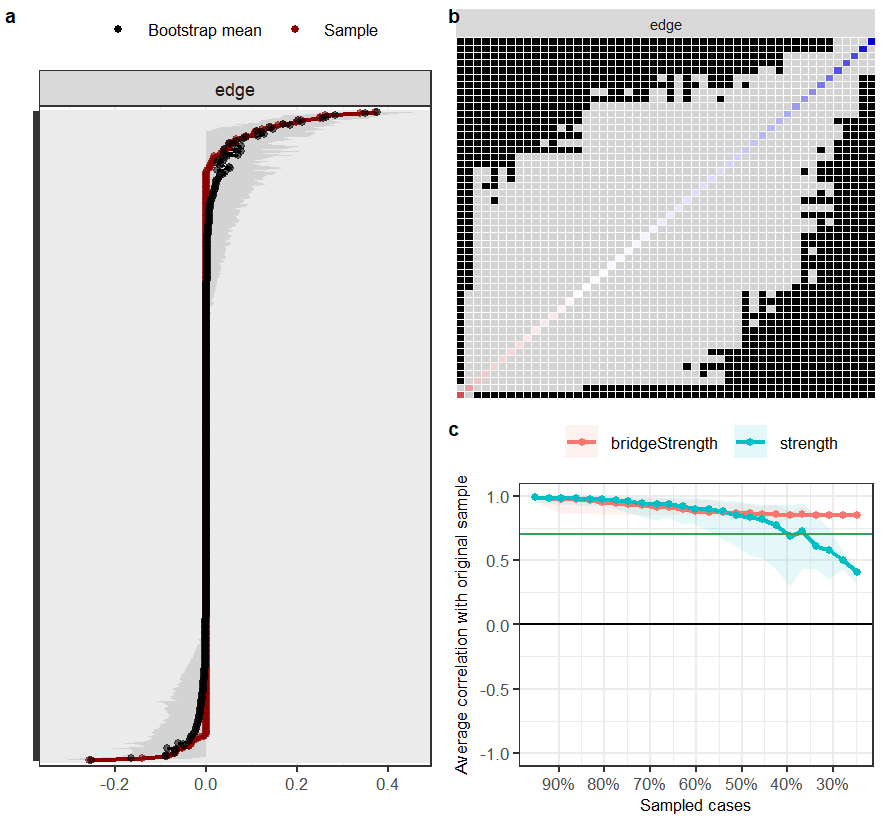


**Figure S11** As in figure S4 for the younger girls (10-17 years old) group


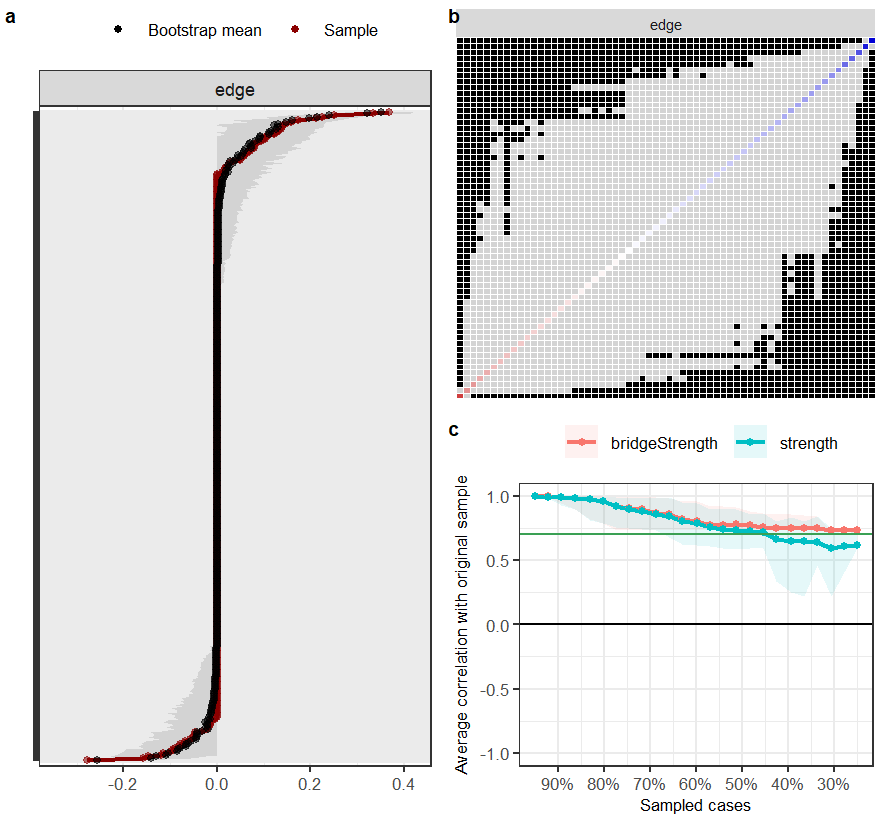


**Figure S12** As in figure S4 for the older girls (17-24) group

### 2.2.2 Consistency of community structure

To assess the consistency of the dimensional structure, we used the bootEGA function from the EGAnet package with 2000 parametric bootstrap samples. We report both the resulting network and its communities (which closely resembles the one estimated with qgraph and presented in Figures 1 and 3 of the main manuscript) and the bootstrap stability results.

Regarding structural consistency, community 1 (red) displayed a stability value of 0.342, community 2 (blue) 0.420, community 3 (green) 0.556, community 4 (orange) 0.458, and community 5 (yellow) 0.680.

Item-wise dimension stability (see fig. S9) showed high consistency for several constructs, including private self-consciousness (1.000), emotional awareness (1.000), body listening (1.000), and body trusting (0.996). In contrast, some variables showed lower replication across bootstrap samples, such as chronic pain (0.470), body esteem attribution (0.510), resistance to peer influence (0.680), and not-distracting (0.676), suggesting that their community clustering may be less stable and likely accounts for variation across models. Item-wise consistency is presented in figure s8.

Notably, items with lower stability values (< 0.70) were not those identified as bridge items or those with the strongest edges. The low stability of body esteem attribution, in particular, helps explaining its reallocation across models. Overall, the reduced stability of certain items largely accounts for the lower stability of some dimensions. Nevertheless, the bootstrap results support the robustness of the main findings, as discussed in the primary manuscript.


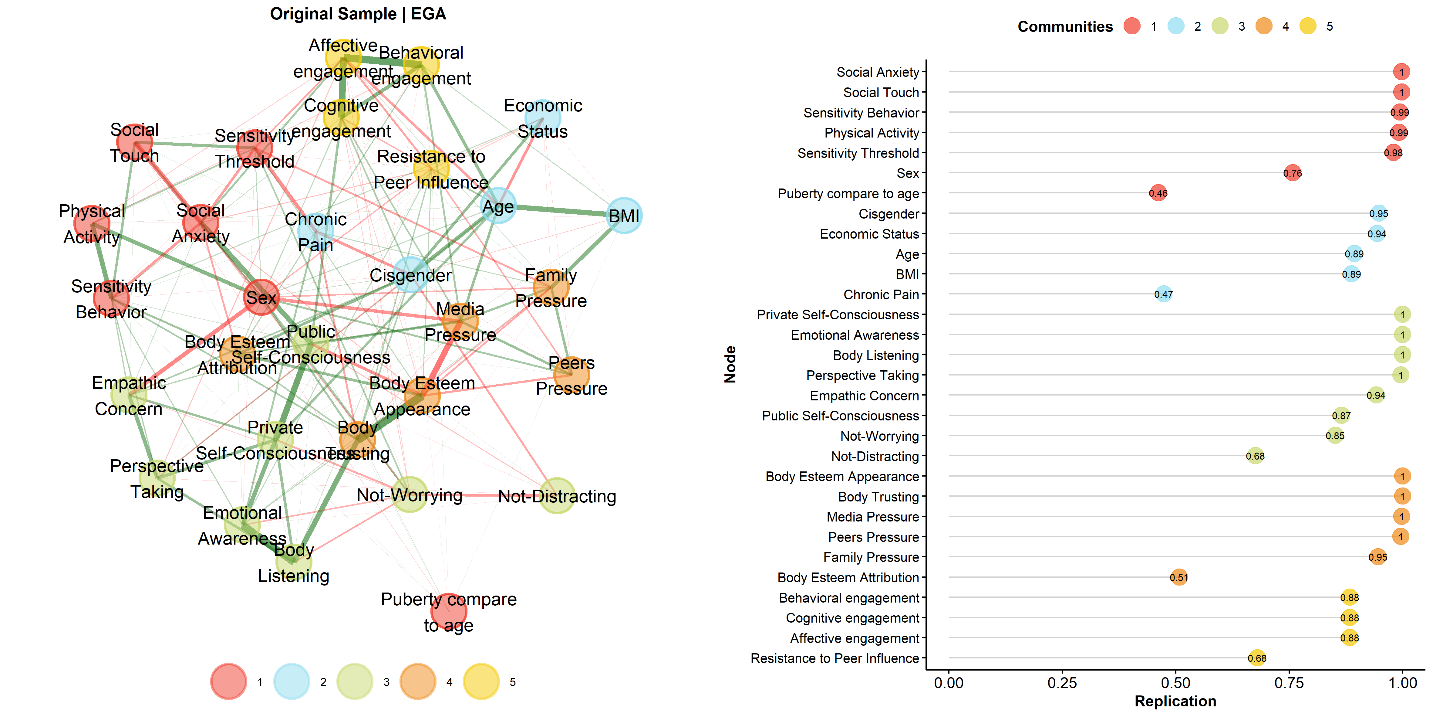


***Figure S13****. Item-wise dimensional consistency of each node in the network based on bootstrap analysis.*

## 2.3 Descriptive statistics and t-test comparison between subgroups

### 2.3.1 Sex comparison

Tables S3 and S4 show the descriptive statistics of the different measures for the girls network. Tables S5 and S6 show the descriptive statistics of the different measures for the boys network.

Table S3 : Descriptive statistics of the continuous measures in girls.

| Measures | Minimum | Maximum | Mean | SD | skew | kurtosis |
| --- | --- | --- | --- | --- | --- | --- |
| Age | 121.00 | 303.00 | 205.79 | 39.18 | -0.01 | 2.29 |
| BMI | 13.79 | 41.38 | 21.51 | 3.95 | 1.17 | 5.66 |
| Puberty | 1.00 | 4.00 | 3.37 | 0.64 | -1.24 | 4.13 |
| Private Self-Consciousness | 1.38 | 5.00 | 3.72 | 0.69 | -0.56 | 3.23 |
| Public Self-Consciousness | 1.00 | 5.00 | 3.61 | 0.88 | -0.55 | 2.83 |
| Social Anxiety | 1.00 | 5.00 | 3.31 | 0.93 | -0.25 | 2.38 |
| Perspective Taking | 1.00 | 5.00 | 3.40 | 0.72 | -0.30 | 2.83 |
| Empathic Concern | 1.43 | 5.00 | 3.98 | 0.68 | -0.69 | 3.35 |
| Behavioral engagement | 1.00 | 5.00 | 4.04 | 1.14 | -1.31 | 3.82 |
| Cognitive engagement | 1.00 | 5.00 | 2.72 | 1.11 | 0.09 | 2.04 |
| Affective engagement | 1.00 | 5.00 | 2.50 | 1.01 | 0.27 | 2.26 |
| Body Esteem Appearance | 1.00 | 5.00 | 3.11 | 0.92 | -0.17 | 2.28 |
| Body Esteem Attribution | 1.00 | 5.00 | 2.96 | 0.71 | -0.05 | 3.21 |
| Body Esteem Weight | 1.00 | 5.00 | 3.11 | 1.14 | -0.18 | 1.93 |
| Family Pressure | 1.00 | 5.00 | 2.61 | 1.10 | 0.31 | 2.15 |
| Peers Pressure | 1.00 | 4.50 | 2.16 | 0.86 | 0.40 | 2.33 |
| Media Pressure | 1.00 | 5.00 | 3.35 | 1.04 | -0.22 | 2.21 |
| Noticing body sensations | 1.00 | 5.00 | 3.43 | 0.81 | -0.30 | 2.79 |
| Not-Distracting | 1.00 | 5.00 | 2.70 | 0.93 | 0.13 | 2.48 |
| Not-Worrying | 1.00 | 5.00 | 2.88 | 0.92 | 0.02 | 2.45 |
| Emotional Awareness | 1.00 | 5.00 | 3.45 | 0.93 | -0.43 | 2.60 |
| Body Listening | 1.00 | 5.00 | 2.62 | 1.03 | 0.36 | 2.46 |
| Body Trusting | 1.00 | 5.00 | 3.26 | 1.01 | -0.16 | 2.48 |
| Social Touch | 1.00 | 4.71 | 2.75 | 0.81 | -0.03 | 2.40 |
| Sensitivity Threshold | 1.57 | 4.36 | 3.20 | 0.50 | -0.26 | 2.80 |
| Sensitivity Behavior | 1.56 | 4.67 | 3.39 | 0.55 | -0.13 | 2.73 |
| Resistance to Peer Influence | 1.00 | 4.00 | 3.11 | 0.60 | -0.90 | 4.18 |
| Puberty compared to age | -1.50 | 1.13 | 0.00 | 0.40 | -0.59 | 3.77 |

Table S4 : Descriptive statistics of the ordinal measures in the girls.

| Measures | Level.1 | Level.2 | Level.3 | Level.4 | Level.5 |
| --- | --- | --- | --- | --- | --- |
| Physical Activity NB | N = 241 | N = 98 | N = 109 | N = 57 |  |
| Physical Activity Freq | 0.48 \% | 0.19 \% | 0.22 \% | 0.11 \% |  |
| Chronic Pain NB | N = 329 | N = 62 | N = 59 | N = 31 | N = 24 |
| Chronic Pain Freq | 0.65 \% | 0.12 \% | 0.12 \% | 0.06 \% | 0.05 \% |
| Economic Status NB | N = 40 | N = 122 | N = 257 | N = 86 |  |
| Economic Status Freq | 0.08 \% | 0.24 \% | 0.51 \% | 0.17 \% |  |

Table S5 : Descriptive statistics of the continuous measures in boys.

| Measures | Minimum | Maximum | Mean | SD | skew | kurtosis |
| --- | --- | --- | --- | --- | --- | --- |
| Age | 129.00 | 294.00 | 208.46 | 41.21 | 0.01 | 2.05 |
| BMI | 12.33 | 34.47 | 21.00 | 3.40 | 0.52 | 3.57 |
| Puberty | 1.00 | 4.00 | 3.00 | 0.77 | -0.48 | 2.35 |
| Private Self-Consciousness | 1.50 | 5.00 | 3.53 | 0.73 | -0.37 | 2.49 |
| Public Self-Consciousness | 1.43 | 5.00 | 3.38 | 0.87 | -0.20 | 2.39 |
| Social Anxiety | 1.00 | 5.00 | 2.81 | 0.93 | 0.02 | 2.43 |
| Perspective Taking | 1.33 | 5.00 | 3.33 | 0.72 | -0.19 | 2.70 |
| Empathic Concern | 1.33 | 5.00 | 3.60 | 0.83 | -0.45 | 2.85 |
| Behavioral engagement | 1.00 | 5.00 | 3.62 | 1.25 | -0.78 | 2.52 |
| Cognitive engagement | 1.00 | 5.00 | 2.39 | 1.07 | 0.46 | 2.35 |
| Affective engagement | 1.00 | 5.00 | 2.18 | 0.98 | 0.69 | 3.03 |
| Body Esteem Appearance | 1.00 | 5.00 | 3.48 | 0.80 | -0.58 | 3.19 |
| Body Esteem Attribution | 1.00 | 5.00 | 3.09 | 0.74 | -0.08 | 3.26 |
| Body Esteem Weight | 1.00 | 5.00 | 3.82 | 0.88 | -0.97 | 3.49 |
| Family Pressure | 1.00 | 5.00 | 2.12 | 0.89 | 0.60 | 2.76 |
| Peers Pressure | 1.00 | 4.75 | 2.24 | 0.87 | 0.50 | 2.70 |
| Media Pressure | 1.00 | 5.00 | 2.74 | 0.93 | 0.44 | 2.70 |
| Noticing body sensations | 1.00 | 5.00 | 3.48 | 0.82 | -0.48 | 3.46 |
| Not-Distracting | 1.00 | 5.00 | 2.74 | 0.97 | 0.21 | 2.54 |
| Not-Worrying | 1.00 | 5.00 | 3.24 | 0.83 | -0.21 | 2.93 |
| Emotional Awareness | 1.00 | 5.00 | 3.37 | 0.96 | -0.41 | 2.72 |
| Body Listening | 1.00 | 5.00 | 2.55 | 1.00 | 0.39 | 2.60 |
| Body Trusting | 1.00 | 5.00 | 3.76 | 0.95 | -0.73 | 3.23 |
| Social Touch | 1.00 | 4.71 | 3.10 | 0.73 | -0.31 | 2.70 |
| Sensitivity Threshold | 1.70 | 4.64 | 3.44 | 0.53 | -0.31 | 3.17 |
| Sensitivity Behavior | 1.78 | 4.67 | 3.47 | 0.53 | -0.07 | 2.74 |
| Resistance to Peer Influence | 1.00 | 4.00 | 3.04 | 0.65 | -0.98 | 4.34 |
| Puberty compared to age | -1.48 | 1.40 | 0.00 | 0.46 | -0.38 | 3.33 |

Table S6 : Descriptive statistics of the ordinal measures in the boys.

| Measures | Level.1 | Level.2 | Level.3 | Level.4 | Level.5 |
| --- | --- | --- | --- | --- | --- |
| Physical Activity NB | N = 71 | N = 28 | N = 63 | N = 67 |  |
| Physical Activity Freq | 0.31 \% | 0.12 \% | 0.28 \% | 0.29 \% |  |
| Chronic Pain NB | N = 173 | N = 27 | N = 19 | N = 5 | N = 5 |
| Chronic Pain Freq | 0.76 \% | 0.12 \% | 0.08 \% | 0.02 \% | 0.02 \% |
| Economic Status NB | N = 16 | N = 60 | N = 119 | N = 34 |  |
| Economic Status Freq | 0.07 \% | 0.26 \% | 0.52 \% | 0.15 \% |  |

We compared each measure between boys and girls using a Student t-test. There are significant differences in 16 cases. Among those we note that Body trust and body appearance are both significantly higher in boys than girls (figure S7), but their link is significantly stronger in the girls’ network.


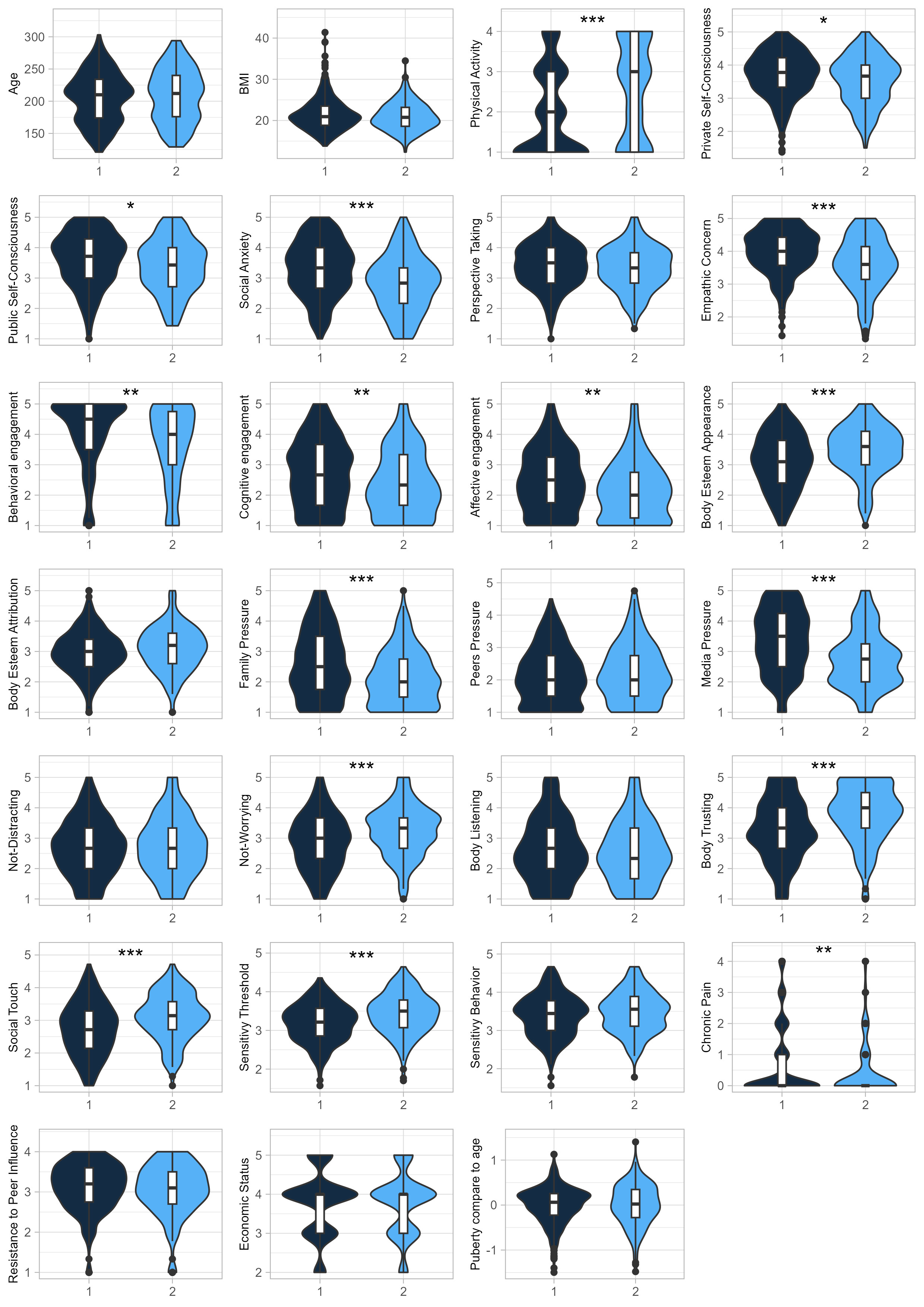


**Figure S14** For each measure the distribution amongst boys (dark blue) and girls (light blue) is plotted. They are compared using Student t-test and corrected for multiple comparisons using Bonferoni correction. ***: p < .001; **: p < .01; *: p < .05 . p value are computed usign parametric t-test and corrected for multiples comparisons.

### 2.3.2 Age Comparison

Tables S7 and S8 show the descriptive statistics of the different measures for the young girls network. Tables S9 and S10 show the descriptive statistics of the different measures for the older girls network.

Table S7 : Descriptive statistics of the continuous measures and resistance to peer influence for the young girls.

| Measures | Minimum | Maximum | Mean | SD | skew | kurtosis |
| --- | --- | --- | --- | --- | --- | --- |
| Age | 121.00 | 204.00 | 169.59 | 20.92 | -0.21 | 2.22 |
| BMI | 13.79 | 30.48 | 20.16 | 3.20 | 0.41 | 2.77 |
| Puberty | 1.00 | 4.00 | 2.98 | 0.68 | -0.70 | 2.79 |
| Private Self-Consciousness | 1.38 | 5.00 | 3.56 | 0.76 | -0.50 | 3.02 |
| Public Self-Consciousness | 1.00 | 5.00 | 3.43 | 0.99 | -0.39 | 2.37 |
| Social Anxiety | 1.00 | 5.00 | 3.26 | 0.88 | -0.11 | 2.53 |
| Perspective Taking | 1.33 | 5.00 | 3.30 | 0.73 | -0.16 | 2.61 |
| Empathic Concern | 1.71 | 5.00 | 3.88 | 0.67 | -0.42 | 2.92 |
| Behavioral engagement | 1.00 | 5.00 | 3.75 | 1.31 | -0.89 | 2.55 |
| Cognitive engagement | 1.00 | 5.00 | 2.54 | 1.16 | 0.33 | 2.10 |
| Affective engagement | 1.00 | 5.00 | 2.54 | 1.06 | 0.08 | 1.98 |
| Body Esteem Appearance | 1.00 | 5.00 | 3.20 | 1.00 | -0.21 | 2.06 |
| Body Esteem Attribution | 1.00 | 5.00 | 2.87 | 0.74 | 0.08 | 3.02 |
| Body Esteem Weight | 1.00 | 5.00 | 3.27 | 1.19 | -0.32 | 1.88 |
| Family Pressure | 1.00 | 5.00 | 2.44 | 1.10 | 0.39 | 2.13 |
| Peers Pressure | 1.00 | 4.50 | 2.14 | 0.92 | 0.41 | 2.20 |
| Media Pressure | 1.00 | 5.00 | 3.05 | 1.10 | 0.05 | 2.15 |
| Noticing body sensations | 1.00 | 5.00 | 3.41 | 0.83 | -0.41 | 2.86 |
| Not-Distracting | 1.00 | 5.00 | 2.71 | 1.00 | 0.09 | 2.26 |
| Not-Worrying | 1.00 | 5.00 | 2.96 | 0.94 | -0.01 | 2.51 |
| Emotional Awareness | 1.00 | 5.00 | 3.31 | 1.00 | -0.44 | 2.44 |
| Body Listening | 1.00 | 5.00 | 2.48 | 1.02 | 0.49 | 2.67 |
| Body Trusting | 1.00 | 5.00 | 3.25 | 1.08 | -0.17 | 2.38 |
| Social Touch | 1.00 | 4.57 | 2.77 | 0.78 | -0.02 | 2.41 |
| Sensitivity Threshold | 1.57 | 4.36 | 3.13 | 0.51 | -0.08 | 2.59 |
| Sensitivity Behavior | 1.78 | 4.67 | 3.38 | 0.59 | -0.07 | 2.53 |
| Resistance to Peer Influence | 1.00 | 4.00 | 3.00 | 0.63 | -0.66 | 3.52 |
| Puberty compared to age | -1.50 | 1.13 | 0.02 | 0.47 | -0.28 | 2.97 |

Table S8 : Descriptive statistics of the ordinal measures for the young girls.

| Measures | Level.1 | Level.2 | Level.3 | Level.4 | Level.5 |
| --- | --- | --- | --- | --- | --- |
| Physical Activity NB | N = 113 | N = 33 | N = 53 | N = 27 |  |
| Physical Activity Freq | 0.5 \% | 0.15 \% | 0.23 \% | 0.12 \% |  |
| Chronic Pain NB | N = 147 | N = 26 | N = 25 | N = 17 | N = 11 |
| Chronic Pain Freq | 0.65 \% | 0.12 \% | 0.11 \% | 0.08 \% | 0.05 \% |
| Economic Status NB | N = 14 | N = 50 | N = 117 | N = 45 |  |
| Economic Status Freq | 0.06 \% | 0.22 \% | 0.52 \% | 0.2 \% |  |

Table S9 : Descriptive statistics of the continuous measures and resistance to peer influence for the older girls.

| Measures | Minimum | Maximum | Mean | SD | skew | kurtosis |
| --- | --- | --- | --- | --- | --- | --- |
| Age | 205.00 | 303.00 | 235.11 | 22.38 | 0.76 | 2.84 |
| BMI | 15.02 | 41.38 | 22.61 | 4.16 | 1.34 | 5.60 |
| Puberty | 2.20 | 4.00 | 3.69 | 0.36 | -1.35 | 4.74 |
| Private Self-Consciousness | 2.11 | 5.00 | 3.84 | 0.60 | -0.36 | 2.65 |
| Public Self-Consciousness | 1.57 | 5.00 | 3.75 | 0.75 | -0.45 | 2.80 |
| Social Anxiety | 1.00 | 5.00 | 3.34 | 0.96 | -0.35 | 2.29 |
| Perspective Taking | 1.00 | 5.00 | 3.49 | 0.71 | -0.42 | 3.13 |
| Empathic Concern | 1.43 | 5.00 | 4.06 | 0.68 | -0.94 | 3.96 |
| Behavioral engagement | 1.00 | 5.00 | 4.27 | 0.92 | -1.66 | 5.64 |
| Cognitive engagement | 1.00 | 5.00 | 2.87 | 1.04 | -0.06 | 2.10 |
| Affective engagement | 1.00 | 5.00 | 2.46 | 0.98 | 0.45 | 2.59 |
| Body Esteem Appearance | 1.00 | 4.90 | 3.03 | 0.83 | -0.25 | 2.45 |
| Body Esteem Attribution | 1.00 | 5.00 | 3.04 | 0.68 | -0.11 | 3.50 |
| Body Esteem Weight | 1.00 | 5.00 | 2.98 | 1.07 | -0.12 | 2.01 |
| Family Pressure | 1.00 | 5.00 | 2.75 | 1.09 | 0.27 | 2.16 |
| Peers Pressure | 1.00 | 4.25 | 2.18 | 0.81 | 0.40 | 2.44 |
| Media Pressure | 1.00 | 5.00 | 3.60 | 0.92 | -0.29 | 2.21 |
| Noticing body sensations | 1.00 | 5.00 | 3.45 | 0.79 | -0.18 | 2.69 |
| Not-Distracting | 1.00 | 5.00 | 2.69 | 0.88 | 0.16 | 2.68 |
| Not-Worrying | 1.00 | 5.00 | 2.81 | 0.89 | 0.01 | 2.37 |
| Emotional Awareness | 1.00 | 5.00 | 3.56 | 0.85 | -0.29 | 2.44 |
| Body Listening | 1.00 | 5.00 | 2.73 | 1.03 | 0.27 | 2.36 |
| Body Trusting | 1.00 | 5.00 | 3.26 | 0.96 | -0.15 | 2.53 |
| Social Touch | 1.00 | 4.71 | 2.73 | 0.84 | -0.02 | 2.37 |
| Sensitivity Threshold | 1.71 | 4.36 | 3.25 | 0.48 | -0.39 | 3.11 |
| Sensitivity Behavior | 1.56 | 4.56 | 3.40 | 0.51 | -0.17 | 2.89 |
| Resistance to Peer Influence | 1.00 | 4.00 | 3.20 | 0.55 | -1.09 | 5.13 |
| Puberty compared to age | -1.40 | 0.44 | -0.01 | 0.34 | -1.26 | 4.68 |

Table S10: Descriptive statistics of the ordinal measures for the older girls.

| Measures | Level.1 | Level.2 | Level.3 | Level.4 | Level.5 |
| --- | --- | --- | --- | --- | --- |
| Physical Activity NB | N = 128 | N = 65 | N = 56 | N = 30 |  |
| Physical Activity Freq | 0.46 \% | 0.23 \% | 0.2 \% | 0.11 \% |  |
| Chronic Pain NB | N = 182 | N = 36 | N = 34 | N = 14 | N = 13 |
| Chronic Pain Freq | 0.65 \% | 0.13 \% | 0.12 \% | 0.05 \% | 0.05 \% |
| Economic Status NB | N = 26 | N = 72 | N = 140 | N = 41 |  |
| Economic Status Freq | 0.09 \% | 0.26 \% | 0.5 \% | 0.15 \% |  |

We compared each measure between younger and older girls using a Student t-test (Figure S10). Body Trust, Body Esteem Attribution, Social Anxiety, and Sensitivity Behavior are all not significantly different in the two subgroups.


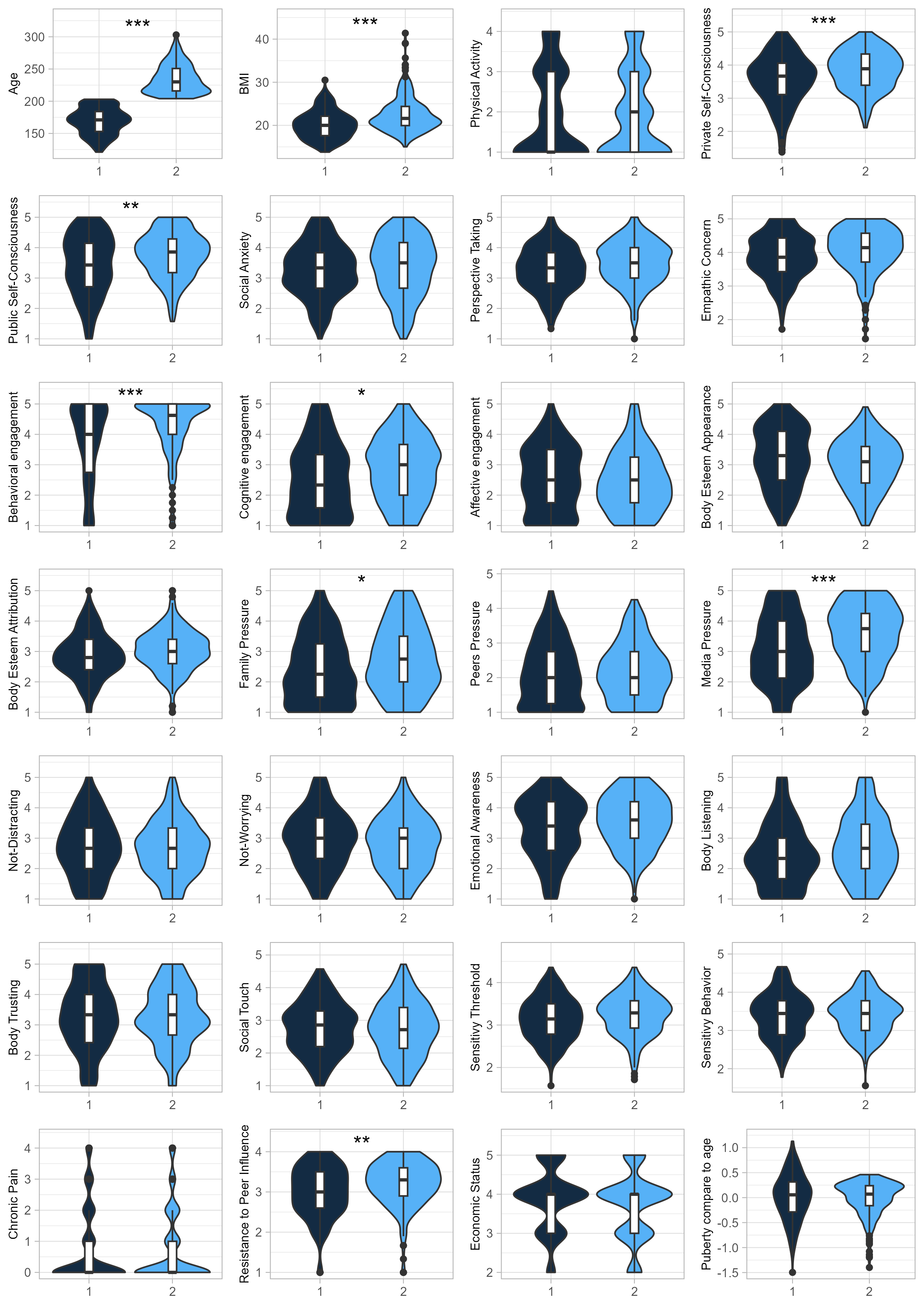


**Figure S15** Age comparison for each measure. For each measure, the distribution amongst young and older girls is plotted. ***: p < .001; **: p < .01; *: p < .05 . p values are computed usind parametric t-test and corrected for multiple comparisons.

# References

Beldo, S. K., Aars, N. A., Christoffersen, T., Furberg, A.-S., Halvorsen, P. A., Hansen, B. H., Horsch, A., Sagelv, E. H., Syed, S. et Morseth, B. (2022). Criterion validity of the Saltin-Grimby Physical Activity Level Scale in adolescents. The Fit Futures Study. *PLOS ONE*, *17*(9), e0273480. <https://doi.org/10.1371/journal.pone.0273480>

Bringmann, L. F., Elmer, T., Epskamp, S., Krause, R. W., Schoch, D., Wichers, M., Wigman, J. T. W. et Snippe, E. (2019). What do centrality measures measure in psychological networks? *Journal of Abnormal Psychology*, *128*(8), 892-903. <https://doi.org/10.1037/abn0000446>

Brown, C. et Dunn, W. (2002). *Adolescent/Adult sensory profile*. Pearson San Antonio, TX, USA:

Buuren, S. V. et Groothuis-Oudshoorn, K. (2011). Mice : Multivariate Imputation by Chained Equations in R. *Journal of Statistical Software*, *45*(3). <https://doi.org/10.18637/jss.v045.i03>

Epskamp, S., Borsboom, D. et Fried, E. I. (2018). Estimating psychological networks and their accuracy: A tutorial paper. *Behavior Research Methods*, *50*(1), 195-212. <https://doi.org/10.3758/s13428-017-0862-1>

Fritz, J., Stochl, J., Fried, E. I., Goodyer, I. M., Van Borkulo, C. D., Wilkinson, P. O. et Van Harmelen, A.-L. (2019). Unravelling the complex nature of resilience factors and their changes between early and later adolescence. *BMC Medicine*, *17*(1), 203. <https://doi.org/10.1186/s12916-019-1430-6>

Grimby, G., Börjesson, M., Jonsdottir, I. H., Schnohr, P., Thelle, D. S. et Saltin, B. (2015). The "Saltin-Grimby Physical Activity Level Scale" and its application to health research. *Scandinavian Journal of Medicine & Science in Sports*, *25 Suppl 4*, 119-125. <https://doi.org/10.1111/sms.12611>

Isoard-Gautheur, S., Ginoux, C., Petit, R., Clavier, V., Dias, D., Sarrazin, P. et Couturier, K. (2023). Relationships between food insecurity, physical activity, detachment from studies, and students’ well-being: A prospective study. *Scandinavian Journal of Medicine & Science in Sports*, *n/a*(n/a). <https://doi.org/10.1111/sms.14361>

Isvoranu, A.-M., Epskamp, S. (2023). Which estimation method to choose in network psychometrics? Deriving guidelines for applied researchers. *Psychological Methods*, 28, 925–946. https://doi.org/10.1037/met0000439

Ni, X., Shao, X., Geng, Y., Qu, R., Niu, G. et Wang, Y. (2020). Development of the Social Media Engagement Scale for Adolescents. *Frontiers in Psychology*, *11*, 701. <https://doi.org/10.3389/fpsyg.2020.00701>

Petersen, A. C., Crockett, L., Richards, M. et Boxer, A. (1988). A self-report measure of pubertal status: Reliability, validity, and initial norms. *Journal of Youth and Adolescence*, *17*(2), 117-133. <https://doi.org/10.1007/BF01537962>

Shirtcliff, E. A., Dahl, R. E. et Pollak, S. D. (2009). Pubertal Development: Correspondence Between Hormonal and Physical Development. *Child Development*, *80*(2), 327-337. <https://doi.org/10.1111/j.1467-8624.2009.01263.x>

Takahashi, M. (2017). Statistical Inference in Missing Data by MCMC and Non-MCMC Multiple Imputation Algorithms: Assessing the Effects of Between-Imputation Iterations. *Data Science Journal*, *16*(0), 37. <https://doi.org/10.5334/dsj-2017-037>

Wilhelm, F. H., Kochar, A. S., Roth, W. T. et Gross, J. J. (2001). Social anxiety and response to touch: Incongruence between self-evaluative and physiological reactions. *Biological Psychology*, *58*(3), 181-202. <https://doi.org/10.1016/S0301-0511(01)00113-2>

Williams, D. R., Rast, P. (2020). Back to the basics: Rethinking partial correlation network methodology. *British Journal of Mathematical and Statistical Psychology,* 73, 187–212. https://doi.org/10.1111/bmsp.12173
